# Interventions to Promote Fundamental Movement Skills in Childcare and Kindergarten: A Systematic Review and Meta-Analysis

**DOI:** 10.1007/s40279-017-0723-1

**Published:** 2017-04-06

**Authors:** Kristin Wick, Claudia S. Leeger-Aschmann, Nico D. Monn, Thomas Radtke, Laura V. Ott, Cornelia E. Rebholz, Sergio Cruz, Natalie Gerber, Einat A. Schmutz, Jardena J. Puder, Simone Munsch, Tanja H. Kakebeeke, Oskar G. Jenni, Urs Granacher, Susi Kriemler

**Affiliations:** 1 Division of Training and Movement Sciences, University of Potsdam, Potsdam, Germany; 2 University of Applied Science for Sport and Management Potsdam of the ESAB, Potsdam, Germany; 3 Epidemiology, Biostatistics and Prevention Institute, University of Zurich, Hirschengraben 84, 8001 Zurich, Switzerland; 4 Endocrinology, Diabetes and Metabolism Service, Centre Hospitalier Universitaire Vaudois (CHUV), Lausanne, Switzerland; 5 Division of Pediatric Endocrinology, Diabetology and Obesity, Centre Hospitalier Universitaire Vaudois (CHUV), Lausanne, Switzerland; 6 Department of Clinical Psychology and Psychotherapy, University of Fribourg, Fribourg, Switzerland; 7 Child Development Center and Children’s Research Center, University Children’s Hospital Zurich, Zurich, Switzerland

## Abstract

**Background:**

Proficiency in fundamental movement skills (FMS) lays the foundation for being physically active and developing more complex motor skills. Improving these motor skills may provide enhanced opportunities for the development of a variety of perceptual, social, and cognitive skills.

**Objective:**

The objective of this systematic review and meta-analysis was to assess the effects of FMS interventions on actual FMS, targeting typically developing young children.

**Method:**

Searches in seven databases (CINAHL, Embase, MEDLINE, PsycINFO, PubMed, Scopus, Web of Science) up to August 2015 were completed. Trials with children (aged 2–6 years) in childcare or kindergarten settings that applied FMS-enhancing intervention programs of at least 4 weeks and meeting the inclusion criteria were included. Standardized data extraction forms were used. Risk of bias was assessed using a standard scoring scheme (Effective Public Health Practice Project—Quality Assessment Tool for Quantitative Studies [EPHPP]). We calculated effects on overall FMS, object control and locomotor subscales (OCS and LMS) by weighted standardized mean differences (SMD_between_) using random-effects models. Certainty in training effects was evaluated using GRADE (Grading of Recommendations Assessment, Development, and Evaluation System).

**Results:**

Thirty trials (15 randomized controlled trials and 15 controlled trials) involving 6126 preschoolers (aged 3.3–5.5 years) revealed significant differences among groups in favor of the intervention group (INT) with small-to-large effects on overall FMS (SMD_between_ 0.46), OCS (SMD_between_ 1.36), and LMS (SMD_between_ 0.94). Our certainty in the treatment estimates based on GRADE is very low.

**Conclusions:**

Although there is relevant effectiveness of programs to improve FMS proficiency in healthy young children, they need to be interpreted with care as they are based on low-quality evidence and immediate post-intervention effects without long-term follow-up.

**Electronic supplementary material:**

The online version of this article (doi:10.1007/s40279-017-0723-1) contains supplementary material, which is available to authorized users.

## Key Points

**Table:** 

Proficiency in fundamental movement skills (FMS) can and should be trained and enhanced at an early age.
In this review, interventions tackling FMS improvement in typically developing young children (aged 2–6 years) show clear beneficial effects on overall FMS, locomotion, and object control skills.
As there is very little confidence in the effect estimates, and the true effect in this study is most likely different (stronger or weaker) from the effect estimate, more high-quality research with reduced bias is needed.

## Introduction

Fundamental movement skills (FMS) are basic abilities and skills of a child to perform an organized series of basic movements that involve various body parts and provide the basis of achieving a high level of motor competence to develop normally, maintain health, and gain athletic excellence [1–5]. FMS is usually classified into basic locomotor skills that enable children to transfer the body in space (e.g. walking, running, jumping, sliding, hopping, and leaping), and object control skills that enable them to manipulate and project objects (i.e., throwing, catching, striking, bouncing, kicking, pulling, and pushing) [6–8]. Although locomotor and object control subscales (LMS and OCS) are reasonably well correlated (*r* = 0.84–0.96) [8], they should be differentiated, given their discrete and independent importance towards predicting health behaviors [9]. FMS are essential to the more specialized and complex skills used in play, games, and sports. Mastery of these basic motor skills that predominantly evolve during the preschool years [8, 10] is an essential part of pleasant participation and a lifelong interest in a physically active lifestyle [11, 12], or even of becoming an elite athlete [3].

Proficiency in FMS is considered critical to achieving and maintaining physical activity [11, 13] and physical fitness [14], preventing obesity [15–17], and developing more complex motor skills for later life [9, 10]. Yet, an increasing number of young children have insufficiently developed FMS [18–20]. Given that FMS are related to lifelong engagement in physical activity that is essential not only to maintain physical health, but likewise to support cognitive and social development during childhood [21], it is important to promote FMS during the first years of life [11]. The acquisition of FMS is not only achieved through natural development and maturation, but also through continuous interaction with a stimulating and supportive social and physical environment including attractive and sufficient space, a stimulating social attitude, as well as a professional instructional approach. This concept is based on a mutual interaction between the biological conditions and the environment that can be seen as a dynamic developmental system of perception and action [22]. This prepares children to engage in a wide and complex range of physical activities [6, 23] that induces adaptive neuro-motor development, and hence FMS [9, 10]. Based on the conceptual models introduced by Stodden et al. [10] and Robinson et al. [9], there is likely a bidirectional interaction between actual FMS and physical activity, with the association also being mediated by perceived FMS [24] and physical fitness [14]. Although important, this mediating role is yet insufficiently studied in young children [9] and therefore not in the scope of this review.

In the past, several reviews have covered the effects of FMS intervention programs on FMS in children. However, those articles either examined healthy school-aged children [25, 26], children with motor disabilities or handicaps [27, 28], or focused on physical activity [29, 30], which is clearly different from FMS. The two reviews with a similar scope to ours included primarily healthy preschool children and were published 5–7 years ago [31, 32]. Although both found that interventions were effective in improving FMS, these articles were methodologically limited and therefore failed to provide solid evidence of the effectiveness of FMS intervention in preschool children. One of these systematic reviews [32] included 17 studies with an intervention duration of 6–24 weeks. Sixty percent of the included studies showed statistically significant intervention effects. However, the authors did not conduct a meta-analysis due to the low methodological quality and the large heterogeneity of the included studies. The other review [31] included 22 studies that were primarily conducted in preschoolers. Findings showed that FMS interventions of 6–35 weeks’ duration produced effect sizes in the range of 0.39–0.45 for overall FMS, OCS, or LMS. However, these authors did not perform any form of quality rating of the included studies. Further, uncontrolled studies were assessed and the meta-analysis was computed based on pre-post values of the intervention groups only.

Due to this gap in the literature, the objective of this systematic review and meta-analysis was to describe and evaluate long-term effects (≥4 weeks) of childcare- and kindergarten-based intervention programs aiming to improve FMS in typically developing children during early childhood (ages 2–6 years). We used the Grading of Recommendations Assessment, Development, and Evaluation System (GRADE) to define certainty in effect estimates for the main outcomes. We further performed subgroup analyses to tease out whether quality, duration of the studies, or the type of teacher (e.g., childcare or kindergarten staff) influenced results. Finally, we performed exploratory analyses to identify interventions that were more effective than others by assessing differences in effect sizes according to type of FMS test used, target groups (e.g., gender), the setting (e.g., childcare versus kindergarten), or intervention characteristics (e.g., duration of the intervention).

## Methods

We conducted and reported this systematic review in accordance with the Preferred Reporting Items for Systematic Reviews and Meta-Analyses (PRISMA) statement [33].

### Literature Search

A librarian experienced in running systematic literature searches carried out a tailored literature search of papers on interventions to promote FMS using CINAHL, Embase, MEDLINE, PsycINFO, PubMed, Scopus and Web of Science from the year of the inception of each database through August 2015 (Electronic Supplementary Material [ESM] Table S1). Based on the PICOS approach [34], our search strategy focused on Population (e.g., children, preschoolers), Intervention (e.g., any type of intervention aiming at increasing FMS and reporting duration, frequency, and dose), Comparator (control group [CON] with usual childcare or kindergarten), Outcome (e.g., motor skills, running, hopping, balance skills), and Study design (e.g., controlled trial [CT], randomized controlled trial [RCT]). A repeated and broadened search approach was conducted after we retrieved a different set of eligible papers in our first searches with strategies that were too focused (e.g., preschoolers versus children, different exclusion criteria based on disease as motor handicaps or chronic disease rather than developmental delay), or too narrow (e.g., search options for the study design such as controlled study versus controlled trial or controlled intervention). Reference lists of included studies and published reviews were screened for additional potentially relevant articles.

### Eligibility Criteria

Eligible studies were either clustered or unclustered CTs or RCTs that enrolled preschool children aged 2–6 years without major health problems or motor handicaps/disability, and assigned them to an intervention (INT) or a control (CON) arm with the specified aim of improving FMS. The intervention needed to take place in a common institutional setting where children of this age range spend their days (e.g., childcare, nursery, preschool, or kindergarten settings), irrespective of whether they belonged to the school or preschool system, with the aim of improving FMS proficiency. The duration of the intervention had to be at least 4 weeks as we were not interested in short-term effects. Further, the trial had to report a standardized motor skill outcome measure (preferably baseline and post-test or pre-post delta values—means, standard deviation [SD], and standard error [SE]) in both arms (INT and CON). We excluded studies not written in English or German, where only the abstract was available, and also trials that enrolled fewer than ten children because of the limited information that we would gain from such small sized studies.

### Study Selection and Data Extraction

Teams of reviewers (CL, KW, LO, NM, SC, SK) worked independently and checked in pairs the eligibility status of identified citations by screening titles, abstracts, and then the full paper. In case of any disagreement, consensus was reached through discussions and also by including a third person. The reviewers used a pretested standardized form to extract information from each eligible study including participants and cluster demographics, intervention details, study methodology, and outcome data. We collected primary outcome data that comprised any measured single motor skill task, composite overall (total FMS), or subscale scores (OCS, LMS) of motor skills. Studies used a wide range of methods to assess FMS (ESM Table S2) and reported a variety of different outcome measures. Other outcome measures (i.e., physical activity and body composition) are not discussed here but are described in Table 1.

**Table 1:** Intervention characteristics of included studies

Study	Design	Target population	Setting; participants (mean age ± SD, years)	Assessment^a^	Intervention program	Overview results^b^
Alhassan et al. [67] United States	Cluster-randomized controlled trial INT and CON: 4 classrooms from 2 preschool centers in each	Ethnic minority preschoolers	Preschool centers INT: *n* = 43 (4.5 ± 0.6), CON: *n* = 28 (4.1 ± 0.6)	FMS: TGMD-2 (LMS) PA: accelerometer BC: height, weight, BMI (*z*-scores) Data collection: 0, 6 months	Duration: 6 months INT: 30 min structured PA lessons 5/week; lessons focusing on one of the skills of the TGMD-2 LMS; training sessions for teachers (total 8 h) CON: 30 min unstructured free play time 5/week; training sessions for teachers (total 2 h)	FMS: leaping INT > CON; remaining tests INT ≈ CON PA: sedentary INT < CON BC: INT ≈ CON
Bellows et al. [36] United States	Cluster-randomized controlled trial INT and CON: 4 Head Start centers each	Preschool-aged children	Head Start centers INT: *n* = 132 (4.4 ± 0.6) CON: *n* = 131 (4.3 ± 0.6)	FMS: PDMS-2 (totFMS, OCS, LMS) PA: pedometers BC: height, weight, BMI (*z*-scores) Data collection: 0, 18 weeks	Duration: 18 weeks INT: 15–20 min structured PA lessons 4/week; lessons including multiple activities focusing on one or a group of skills from one of the three gross motor skill categories (Balance, LMS, OCS) by introducing fictive characters promoting motor skills and food; home material; training sessions for teachers. Additionally, those characters were also part of another nutrition-based study called ‘food friends’, which took place at the same time CON: nutrition-based ‘food friends’ study	FMS: INT > CON PA: INT ≈ CON BC: INT ≈ CON
Bonvin et al. [69] Switzerland	Cluster-randomized controlled trial INT and CON: 29 childcare centers each	Young children aged 2–4 years	Childcare centers INT: *n* = 313 (3.4 ± 0.6) CON: *n* = 335 (3.3 ± 0.6)	FMS: adapted ZNA (totFMS) PA: accelerometer BC: height, weight, BMI Data collection: 0, 9 months	Duration: 9 months INT: 5 workshops for educators providing education in PA; promoting PA to parents via childcare educators; flyers; documentation and support at childcare center through coordinator; focus groups every 2 months; financial support for childcare centers to design activity-friendly spaces CON: regular preschool program	FMS: INT ≈ CON PA: INT ≈ CON BC: INT ≈ CON
Deli^c^ [37] Greece	Controlled trial INT1, INT2 and CON: 1 class each, created out of the study participants	Kindergarten children	Preschool center INT1: *n* = 25 (5.4 ± 0.5) INT2: *n* = 25 (5.5 ± 0.3) CON: *n* = 25 (5.4 ± 0.6)	FMS: TGMD PA: none BC: none Data collection: 0, 10 weeks	Duration: 10 weeks INT1: 35 min structured movement lessons 2/week; lessons in week 1–4 including exercises for body/space awareness, lessons in week 5–10 including locomotor skills INT2: 35 min structured music and movement lessons 2/week focusing on percussion, reaction and creative movements; lessons in week 1–4 including exercises for body/space awareness and for determining personal rhythm, lessons in week 5–7 focusing on movement synchronization to external rhythms, and lessons in week 8–10 combining rhythm and fundamental locomotor skills CON: free-play activities	FMS: INT > CON PA: none BC: none
Derri et al. [70] Greece	Randomized controlled trial INT and CON: 1 group each, created out of the study participants	Preschool children	No information about setting INT: *n* = 35 (N/A) CON: *n* = 33 (N/A) INT and CON: (5.4 ± 0.6)	FMS: TGMD (LMS) PA: none BC: none Data collection: 0, 10 weeks	Duration: 10 weeks INT: 35–40 min structured music and movement lessons 2/week; lessons including body/space awareness, reaction, percussion movements and improvisation skills, and combining rhythm and fundamental locomotor skills CON: 30–40 min free-play activities 2/week	FMS: galloping, leaping, horizontal jump, skipping INT > CON; remaining tests INT ≈ CON PA: none BC: none
Donath et al.^c^ [38] Switzerland	Cluster-randomized controlled trial INT and CON: 3 kindergartens each	Kindergarten children	Kindergartens INT: *n* = 22 (4.4 ± 1.0) CON: *n* = 19 (4.4 ± 1.2)	FMS: TGMD-2 (OCS) PA: none BC: height, weight, BMI Data collection: 0, 6 weeks	Duration: 6 weeks INT: 30 min structured training sessions 2/week; lessons including object control exercises CON: instructed and supervised training 2/week for playing activities by inexperienced instructor; no changes of daily physical and sportive activities	FMS: total sum score, stationary dribbling INT > CON; remaining tests INT ≈ CON PA: none BC: INT ≈ CON
Goodway and Branta [60] United States	Clustered controlled trial INT and CON: 2 preschool classes each	Disadvantaged preschool children	Preschool classes INT: *n* = 31 (4.7 ± 0.3) CON: *n* = 28 (4.7 ± 0.3)	FMS: TGMD (OCS, LMS) PA: none BC: none Data collection: 0, 13 weeks	Duration: 12 weeks INT: 45 min instructional lessons 2/week; lessons including sustained activity (10 min), skill instruction (3 × 10 min), and emphasizing key components of those skills (3 min) CON: typical preschool program including free play time	FMS: INT > CON PA: none BC: none
Goodway et al. [61] United States	Cluster-controlled trial INT and CON: 2 pre-kindergarten classes each	Pre-kindergarten children at risk for DD	Pre-kindergarten INT: *n* = 33 (4.9 ± 0.4) CON: *n* = 30 (5.0 ± 0.4)	FMS: TGMD (OSC, LMS) PA: none BC: none Data collection: 0, 9 weeks	Duration: 9 weeks INT: 35 min instructional sessions 2/week; 3 × 10-min periods of skill instruction, using developmentally and instructionally appropriate practice CON: typical pre-kindergarten curriculum	FMS: INT > CON PA: none BC: none
Hamilton et al. [39] United States	Clustered controlled trial INT: 3 preschool classes CON: 2 preschool classes	Preschool children at risk for DD	Preschool classes INT: *n* = 15 (3.9 ± 0.2) CON: *n* = 12 (4.0 ± 0.3)	FMS: TGMD (OCS) PA: none BC: none Data collection: 0, 8 weeks	Duration: 8 weeks INT: 45 min parent-assisted instructional lessons 2/week; lessons including a minimum of 2 of the 5 object control skills, presented by parents at the center; parent instruction prior to each lesson (15 min); parent orientation meetings previous to study begin (2 × 45 min) CON: regular activity program including movement songs and activities with parents, and opportunities for movement exploration 2/week for 45 min	FMS: INT > CON PA: none BC: none
Hardy et al. [40] Australia	Cluster-randomized controlled trial INT: 15 preschools CON: 14 preschools	Preschool-aged children	Preschools and long-day care centers INT: *n* = 263 (4.4 ± 0.5) CON: *n* = 167 (4.5 ± 0.3)	FMS: TGMD-2 (totFMS, OCS, LMS) PA: none BC: none Data collection: 0, 20 weeks	Duration: 20 weeks INT: 1-day professional workshop for preschool staff (incorporating healthy eating and PA into education program; structural and organizational changes in preschools); resources for preschools (manual; small grant for purchasing activity equipment or support staff to attend training); contact with health promotion professionals CON: preschools provided with written information on sun and road safety	FMS: INT ≈ CON PA: none BC: none
Hashemi et al. [74] Iran	Controlled trial INT and CON: no information about allocation	Preschool girls from non-affluent families without post-graduate education	Kindergartens INT: *n* = 30 (5.1 ± 0.0) CON: *n* = 30 (5.0 ± 0.1)	FMS: TGMD-2 (OCS) PA: none BC: height, weight Data collection: 0, 6 weeks	Duration: 6 weeks INT: 45 min structured lessons 3/week; lessons including warm-up, selected games (i.e. ball dodging), and cool-down CON: regular daily activity	FMS: INT > CON PA: none BC: none
Hurmeric [51] United States	Cluster-randomized controlled trial INT1 and INT2: 3 mixed classes from 1 center CON: 1 group from another center	Preschool children	Head Start centers INT1: *n* = 22 (4.0 ± 0.5) INT2: *n* = 25 (4.1 ± 0.5) CON: *n* = 25 (4.0 ± 0.6)	FMS: TGMD-2 (OCS) PA: none BC: height, weight, BMI, grip strength, body fat Data collection: 0, 8 weeks; follow-up at 12 weeks	Duration: 8 weeks INT1: 30 min structured movement lessons 2/week; lessons including warm-up, instructions for two object control skills (2 × 12 min), and closure activities INT2: same intervention as INT1; additional 10–15 min movement lesson at home conducted by primary caregiver with lesson plan, instructions and standardized equipment provided; workshop prior to intervention CON: regular Head Start curriculum, including outdoor and large muscle activities	FMS: INT1 ≈ INT2 > CON PA: none BC: none
Ignico [41] United States	Clustered controlled trial INT and CON: 1 kindergarten class each	Kindergarten children	Elementary school INT: *n* = 15 (N/A) CON: *n* = 15 (N/A)	FMS: TGMD (totFMS) PA: none BC: none Data collection: 0, 10 weeks	Duration: 10 weeks INT: 28 min structured training sessions 5/week; lessons including 3 stations CON: regular activities, including 20–25 min free play time	FMS: INT > CON PA: none BC: none
Iivonen et al. [54] Finland	Clustered controlled trial INT and CON: 2 classes from 2 preschools each Distinction of sex	Preschool children	Preschools INT: *n* = 39 (N/A) CON: *n* = 35 (N/A) INT and CON: (4.6 ± 0.1)	FMS: adapted APM Inventory (OCS) PA: none BC: none Data collection: 0, 4, 8 months, Follow-up at 11 months	Duration: 8 months INT: 45 min physical education lessons 2/week; lessons according to the Physical Education Curriculum (PEC) of the Early Steps Project [119] CON: 60 min unstructured physical education lesson 1/week	FMS: INT ≈ CON PA: none BC: none
Jones et al. [75] Australia	Cluster-randomized controlled trial INT and CON: 1 class from 1 childcare center each	Preschool children	Childcare centers INT: *n* = 52 (N/A) CON: *n* = 45 (N/A) INT and CON: (4.1 ± N/A)	FMS: TGMD-2 (totFMS) PA: accelerometer BC: height, weight, BMI Data collection: 0, 20 weeks	Duration: 20 weeks INT: 20 min structured PA lessons 3/week; lessons focusing on one motor competency each week; theoretical and practical workshops (4 × 30 min) for the staff; specific equipment provided to childcare CON: usual program, including designated time outside for free play	FMS: INT > CON PA: INT > CON BC: none
Kelly et al. [42] United States	Clustered controlled trial INT: 2 groups from 1 preschool CON: 1 group from another preschool	Preschool children	Motor Development Clinic (INT), Preschool (CON) INT: *n* = 21 (4.4 ± 0.7) CON: *n* = 26 (4.2 ± 0.7)	FMS: MEAP Test PA: none BC: none Data collection: 0, 6, 12 weeks	Duration: 12 weeks INT: 50 min physical education instruction 2/week; lessons including free play (5 min), introductory activities (8 min), instructional activities (30 min) and summary activities (7 min) CON: daily periods of supervised free play on well-equipped playground; no formal instruction in physical education	FMS: INT ≈ CON PA: none BC: none
Krombholz^c^ [43] Germany	Clustered controlled trial INT and CON: 11 childcare centers each	Preschool children	Childcare centers INT: *n* = 211 (4.6 ± 0.6) CON: *n* = 217 (4.5 ± 0.7)	FMS: MoTB 3–7 (totFMS) PA: none BC: height, weight, BMI, body fat Data collection: 0, 11, 20 months	Duration: 20 months INT: 45 min physical education session 1/week; Additional 20 min PA on the other days; lesson contents free to choose; raise awareness and train competency of educators CON: usual curriculum, including 45 min physical education session 1/week	FMS: INT ≈ CON PA: none BC: INT ≈ CON
Piek et al.^c^ [44] Australia	Cluster-randomized controlled trial INT and CON: 6 schools each	Young children aged 4–6 years from low socioeconomic area	Primary schools INT: *n* = 254 (N/A) CON: *n* = 196 (N/A) INT and CON: (5.4 ± 0.3)	FMS: BOT-2SF, MABC-2 (totFMS) PA: none BC: height, weight, BMI (*z*-score), waist circumference Data collection: 0, 6 months; follow-up at 18 months	Duration: 6 months INT: 30 min PA lessons 4/week; lessons including different modules of the Animal Fun Program (body management, locomotion, object control, etc.); 1-day training course for teachers prior to intervention CON: normal curriculum	FMS: INT ≈ CON PA: none BC: none
Puder et al. [71] Switzerland	Cluster-randomized controlled trial INT and CON: 20 preschool classes each from a total of 30 preschools in 2 different country regions	Preschool children from an area with high proportion of migrants	Preschool INT: *n* = 342 (5.2 ± 0.6) CON: *n* = 310 (5.2 ± 0.6)	FMS: shuttle run (20 m), obstacle course, balance beam and platform PA: accelerometer BC: height, weight, BMI, body fat, waist circumference Data collection: 0, 9 months	Duration: 9 months INT: 45 min PA lessons 4/week; lessons including mainly aerobic exercises in/around the school and in the gym; home material; nutritional intervention CON: regular school curriculum, including 45 min PA in the gym 1/week in both regions, and another 45 min rhythmic lesson in the other region	FMS: shuttle run, obstacle course INT > CON; remaining tests INT ≈ CON PA: INT > CON BC: body fat, waist circumference INT < CON; remaining tests INT ≈ CON
Reilly et al. [45] Scotland	Cluster-randomized controlled trial INT and CON: 18 nurseries each	Young children	Nurseries INT: *n* = 268 (4.2 ± 0.3) CON: *n* = 277 (4.1 ± 0.3)	FMS: MABC (totFMS) PA: accelerometer BC: height, weight, BMI (SD score) Data collection: 0, 6 months; follow-up at 12 months	Duration: 24 weeks INT: 30 min PA lessons 3/week; lessons intending to increase PA levels of children and meet the requirements of the ‘physical development and movement’ component of the nursery curriculum of Scotland; training sessions for nurses (3×); resource pack of materials for home based intervention (health education leaflets); posters displayed at nurseries for 6 weeks CON: usual curriculum, with the head teachers agreeing not to enhance physical development and movement curriculum	FMS: INT > CON PA: moderate-vigorous INT > CON; remaining measures INT ≈ CON BC: INT ≈ CON
Robinson and Goodway [55] United States	Cluster-controlled trial INT: 1 Head Start center CON: 1 Head Start center	Preschool children at risk for DD	Head Start centers: INT1/2: *n* = 77 (3.9 ± 0.6) CON: *n* = 40 (4.0 ± 0.4)	FMS: TGMD-2 (OCS) PA: none BC: none Data collection: 0, 9 weeks; follow-up at 9 weeks	Duration: 9 weeks INT: 30 min motor skill intervention 2/week ‘low autonomy’ (INT1) or ‘mastery motivational climate’ (INT2); warm-up activity (2–3 min), motor skill instruction for OC skills (24 min), closure activity (2–3 min), typical Head Start curriculum; +30 min unstructured recess 2/week CON: typical Head Start curriculum; 30 min unstructured recess 2/week	FMS: INT(INT1/2) > CON PA: none BC: none
Roth et al. [72] Germany	Cluster-randomized controlled trial INT: 21 preschools CON: 20 preschools	Preschool children	Preschools INT: *n* = 368 (4.7 ± 0.6 CON: *n* = 341 (4.7 ± 0.5)	FMS: Single items (obstacle course, standing long jump, balancing on one foot, jumping to and fro sideways) − composite *z*-score (totFMS) PA: accelerometer BC: height, weight, BMI (*z*-score), blood pressure, body fat Data collection: 0, 6, 11 months; follow-up at 13–15 months	Duration: 11 months INT: 30 min PA lessons 5/week; lessons including exercises to enhance coordinative skills and perception; manual, collection of games, and exercises for preschools; PA homework cards 1 or 2/week; letters comprising games/exercises for holidays CON: routine schedule, including common daily activity and weekly PA class	FMS: INT > CON, 1-leg stance, standing long jump, lateral jump INT > CON; obstacle course INT ≈ CON PA: INT ≈ CON BC: body fat INT < CON; remaining tests INT ≈ CON
Tsapakidou et al. [46] Greece	Controlled trial INT and CON: 3 kindergartens together	Children aged 3.5–5 years	Nursery schools INT: *n* = 49 (N/A) CON: *n* = 49 (N/A), INT and CON: (3.5–5)	FMS: TGMD-2 (LMS) PA: none BC: none Data collection: 0, 2 months	Duration: 2 months INT: 30–40 min physical education lessons 2/week; lessons including exercises to raise body awareness, rhythm, coordinative skills and creativity to develop basic motor skills CON: daily schedule	FMS: INT > CON PA: none BC: none
Valentini [62] United States	Controlled trial INT and CON: 1 early education center together	Low motor skill functioning children	Early education center INT: *n* = 38 (5.1 ± 0.3) CON: *n* = 29 (5.3 ± 0.5)	FMS: TGMD (OCS, LMS) PA: none BC: none Data collection: 0, 12 weeks; follow-up at 9 months	Duration: 12 weeks INT: 35 min motor skill lessons 2/week; lessons including introduction, motor skill instruction and practice (30 min), and closure, according to TARGET structure [120] CON: regular curriculum	FMS: LMS INT > CON, OCS INT ≈ CON PA: none BC: none
Venetsanou and Kambas [73] Greece	Controlled trial No information about allocation	Preschool children	Kindergarten INT: *n* = 28 (N/A) CON: *n* = 38 (N/A) INT and CON: (5.0 ± 0.5)	FMS: MOT4-6 (totFMS) PA: none BC: none Data collection: 0, 20 weeks	Duration: 20 weeks INT: 45 min musical movement lessons 2/week; lessons including percussive movements and rhythmical locomotion (i.e. singing games, playing percussion instruments) CON: regular kindergarten curriculum activities	FMS: INT > CON PA: none BC: none
Vidoni et al. [68] United States	Cluster-randomized controlled trial INT and CON: 1 class of the same daycare center in each	Preschool children	Daycare center INT: *n* = 18 (N/A) CON: *n* = 15 (N/A) INT and CON: (4.5 ± N/A)	FMS: BOT-2SF (totFMS) PA: none BC: none Data collection: 0, 11 weeks	Duration: 11 weeks INT: 30 min structured PA program 5/week; lessons including circuit training and exercises based on the MAZE approach [121] CON: regular day-care center schedule, including 30 min unstructured PA 5/week	FMS: INT > CON PA: none BC: none
Wang [47] Taiwan	Controlled trial INT and CON: 1 group from the same preschool in each	Preschool children	Preschool INT: *n* = 30 (N/A) CON: *n* = 30 (N/A) INT and CON: (3–5)	FMS: PDMS-2 PA: none BC: none Data collection: 0, 6 weeks	Duration: 6 weeks INT: 30 min creative movement lessons 2/week; lessons including exploring, developing and creating different movements in relation to dancing CON: unstructured free play	FMS: LMS INT > CON; remaining tests INT ≈ CON PA: none BC: none
Weiss et al. [48] Germany	Controlled trial 1 group from the same kindergarten in each	Kindergarten children	Kindergarten INT: *n* = 24 (4.7 ± N/A) CON: *n* = 22 (4.9 ± N/A)	FMS: MOT4-6 PA: none BC: height, weight, BMI Data collection: 0, 6 months	Duration: 6 months INT: 60 min back training lessons 1/week; lessons including a variety of games in combination with different material CON: usual kindergarten schedule including regular PA lessons	FMS: INT > CON PA: none BC: none
Yin et al. [52] United States	Clustered controlled trial INT1: 14 classes in 2 centers INT2: 5 classes in 1 center CON: 6 classes in 1 center	Preschool-aged children	Head Start centers INT1: *n* = 179 (4.1 ± 0.6) INT2: *n* = 80 (4.2 ± 0.5) CON: *n* = 97 (4.1 ± 0.5)	FMS: LAP-3 (totFMS) PA: pedometer BC: height, weight, BMI (*z*-score) Data collection: 0, 18 weeks; Additional 3 mid-study tests	Duration: 18 weeks INT1: 30–45 min structured and unstructured outdoor lessons 5/week; lessons including gross motor skills teaching and dance instruction; supplemental classroom activities; healthy eating promotion INT2: same intervention as INT1; additional take-home activities, parent obesity education and family support monitoring for healthy eating and PA CON: regular schedule, including unstructured free play on the playground 5/week	FMS: INT1 > CON, INT2 > CON PA: INT1 > CON, INT2 > CON BC: BMI INT1 ≈ CON, INT2 < CON
Zask et al. [49] Australia	Cluster-randomized controlled trial INT: 18 preschools CON: 13 preschools	Children aged 3–6 years	Preschools INT: *n* = 273 (N/A) CON: *n* = 142 (N/A) INT and CON: (4.6 ± 0.6)	FMS: TGMD-2 (totFMS, LMS) PA: none BC: height, weight, BMI (*z*-score), waist circumference Data collection: 0, 10 months	Duration: 10 months INT: 25–30 min structured FMS development lessons 2/week; lessons including warm-up (5 min), games in groups (15–20 min), and cool-down (5 min); small grant for equipment; playground review to encourage more active behavior; workshops and monthly newsletter for parents; healthy eating intervention CON: regular curriculum	FMS: INT > CON PA: none BC: BMI, waist circumference INT < CON

*AMP* Alle kouluikäisten lasten PsykoMotoriset taidot, *BC* body composition, *BMI* body mass index, *BOT-2SF* Bruininks-Oseretsky test of motor proficiency—version 2 Short Form, *CON* control group, *DD* developmental delay, *FMS* fundamental movement skills, *INT* intervention group, *LAP-3* Learning Achievement Profile 3rd edition, *LMS* locomotor subscale, *MABC-2* Movement assessment battery for children-version 2, *MEAP* Michigan Educational Assessment Program, *MOT4-6* Motorik test for 4- to 6-year-old children, *MoTB3-7* motor test battery, *N/A* not available, *OCS* object control subscale, *PA* physical activity, *PDMS-2* Peabody Development Motor Scale—2nd edition, *SD* standard deviation, *TGMD-2* Test of Gross Motor Development—2nd edition, *totFMS* total fundamental movement skill score, *ZNA* Zurich Neuromotor Assessment

^a^Electronic Supplementary Material Table S2 gives an overview of all used FMS test batteries within included studies

^b^For detailed information see Electronic Supplementary Material Table S5; results depicted are only between groups post-intervention provided from studies; for </>, there is a significant difference; for ≈, there is no significant difference

^c^Number of participants and mean age ± SD in years only available for post-test period

### Risk of Bias Assessment

The reviewers assessed the risk of bias of each eligible study using a slightly adapted version of the established ‘Effective Public Health Practice Project Quality Assessment Tool for Quantitative Studies’ (EPHPP) that has been proven valid in assessing Public Health interventions [35] (ESM Table S3). This quality assessment tool rates study procedures as ‘strong’, ‘moderate’, or ‘weak’ using eight scales (selection bias, study design, confounders, blinding, data collection methods, withdrawal/dropouts, intervention integrity, and analyses). The same procedure was always applied. That is, two reviewers from a group of four (CL, LO, NM, SK) independently scored the items for each study as ‘strong’, ‘moderate’, or ‘weak’. In cases of disagreement, consensus was reached by discussion or third party arbitration. We provided an overall ‘strong’ or ‘high quality’ score if no ‘weak’ item score existed and at least four of the eight items were ‘strong’. An overall ‘moderate quality’ score was provided with only one ‘weak’ item score and otherwise only ‘strong’ and ‘moderate’ item scores. The remaining studies were overall rated ‘weak’ or ‘low quality’. The reviewers were not blinded to names of authors, institutions, journal, or the outcomes of the trials.

### Missing Data

We contacted the authors of fourteen studies [36–49] to obtain missing information about the FMS assessments (means of standard or raw scores of single FMS items, OSC, LMS, total scores, SD, and number of participants who took part in INT and CON) to be able to conduct our meta-analysis. Of those, six authors answered [36, 38, 40, 43, 44, 49] and provided detailed information on the requested data. One author answered but could not help [39], and seven authors [37, 41, 42, 45–48] did not respond to our repeated requests. Of those, three studies [41, 45, 46] provided total FMS scores in the original article that could be included in some, but not all meta-analytical calculations. The other four studies [37, 42, 47, 48] did not provide any missing data (mean and SD for single item, subscale, or total FMS scores) and therefore results for meta-analyses were not available. However, these studies reported sufficient descriptive and analytical results to be included in this review.

### Meta-Analyses

Data were extracted for meta-analyses (KW) and checked for accuracy (CL). Studies that provided the number of participants, measures of baseline and post-test values (means and SD or SE) [50] for total FMS proficiency (total FMS score), subscales or single motor skill items were included. Post-intervention values were taken for meta-analyses. We chose the INT that focused on interventions taking place in the childcare or kindergarten setting if more than one INT was included [37, 51, 52]. Outcome data of total FMS proficiency and subscales were pooled after conversion to the most familiar and most used instrument (TGMD-2 [Test of Gross Motor Development—2nd edition]) to enhance interpretability of meta-analyses results [53]. Because of scarce subgroup data (e.g., for gender [49, 54], motivational climates [55]), these groups were combined for the meta-analysis of total FMS scores [56].

To verify the effectiveness of FMS intervention programs in childcare and kindergarten settings, we computed between-group standardized mean differences as SMD_between_ = (mean post-test value in INT group − mean post-test value in CON group)/pooled variance to report the average treatment effect [50]. We combined SMD_between_ according to random-effect analyses to obtain an overall SMD for included studies that were further weighted for magnitude of the respective SE. SMD_between_ were adjusted for the respective sample size (Hedges’ adjusted *g*) [50] and expressed based on Cohen’s (1988) categorizing values for SMD_within_/SMD_between_ of <0.5 as small, 0.5–0.79 as medium, and ≥0.80 as large effects [57]. Studies that provided insufficient data to be included in meta-analyses, but fulfilled our eligibility criteria, were kept in the review [37, 42, 47, 48].

### Investigation of Heterogeneity, Subgroup and Exploratory Analyses

Heterogeneity between studies was assessed using *I* ^2^ statistics. To explain expected heterogeneity among study results, we defined a set of two a priori hypotheses on which sensitivity analyses of subgroups were performed. First, we hypothesized that, based on social-cognitive theory [58] and the stages of behavioral change [59], an intervention of 6–8 months is the minimum amount of time needed for a sustainable change in behavior, not so much by the children themselves, but by the childcare and kindergarten professionals and the parents who direct the behavior of children at this young age. Second, we hypothesized that the results of trials would be influenced by their methodological quality. Only for this purpose, we compared ‘high quality’ trials based on our quality rating with ‘moderate’ and ‘low quality’ studies, respectively (ESM Table S4), using all studies that reported total FMS, OCS, or LMS scores. For three studies that reported both OCS and LMS scores but no total FMS score [60–62], the subscale scores were combined [63] to calculate the total FMS score; the variance was then determined by using a correlation between OCS and LMS of 1.0 as a conservative approach [8]. For both subgroup analyses (e.g., methodological quality, duration of the intervention) we calculated weighted mean SMD_between_ for the subgroups to test our hypotheses using Review Manager 5.3 (Copenhagen: The Nordic Cochrane Center, The Cochrane Collaboration, 2014). Due to the heterogeneity of FMS assessment tools used in studies, we defined a further posteriori hypothesis that test results would not vary according to the test battery used. As the majority of studies used one specific test (TGMD or TGMD-2), we compared those studies that used either version of this test battery versus those that used another test.

Further exploratory analyses were done to identify interventions that were more effective than others. These included the evaluation of differences in effect sizes according to *target groups* (e.g., focusing on risk populations for developmental delays rather than taking a population approach, differences in gender), the *setting* (e.g., kindergarten or childcare) or *intervention characteristics* (e.g., the use of a theoretical framework on which the intervention was built on, the integration of expert teachers versus the usual childcare or kindergarten teacher, parental involvement).

### Certainty in Treatment Estimates

We used the GRADE approach to categorize certainty in effect estimates for all reported outcomes as high, moderate, low, or very low [64]. Based on this approach, RCTs start as high certainty but can be rated down because of risk of bias, inconsistency, indirectness, imprecision, and publication bias. CTs start as low certainty, but can be upgraded based on large magnitude effects, dose-response results or confounders that likely minimized the effect [65]. The results are presented in GRADE evidence profiles [66] using GRADEproGDT (http://www.guidelinedevelopment.org/).

## Results

### Study Characteristics

**Fig. 1:**
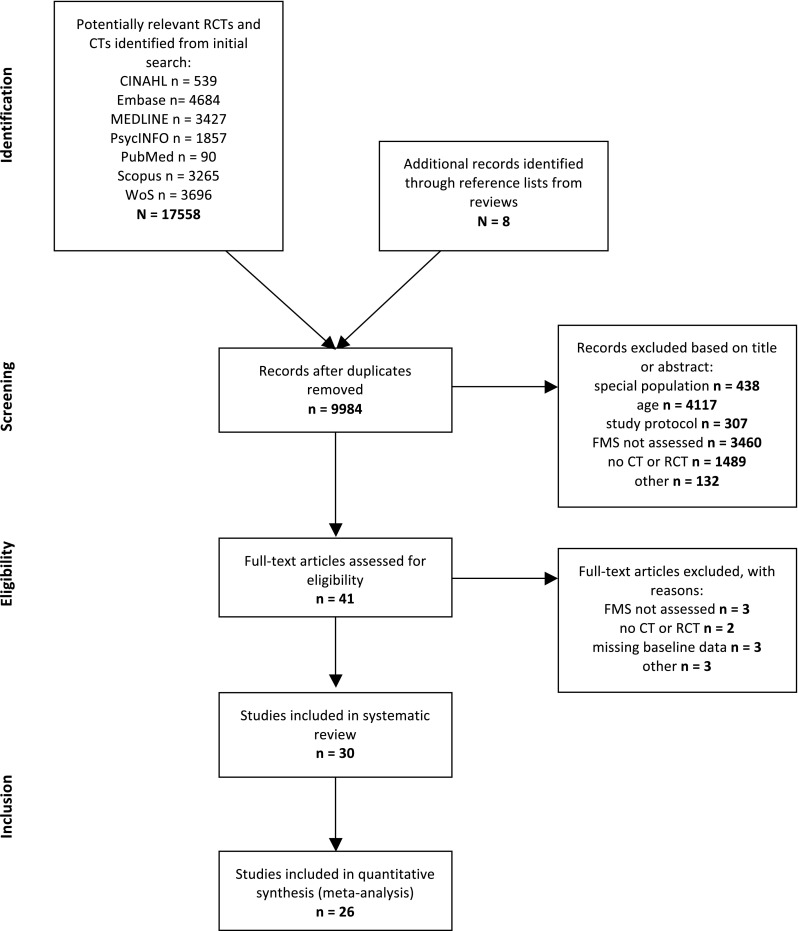
Study flow chart [33]. *CT* controlled trial, *FMS* fundamental movement skills, *RCT* randomized controlled trial, *WoS* Web of Science

Overall, we identified 17,566 unique records, of which we assessed 41 articles for eligibility (Fig. 1). After reviewing the full texts, 30 articles were eligible including 6126 children with an age range of 3.3–5.5 years. All included trials are shown in Table 1. Twelve of the 30 studies were carried out in the US [36, 39, 41, 42, 51, 52, 55, 60–62, 67, 68], 12 in European countries [37, 38, 43, 45, 46, 48, 54, 69–73], and the remainder elsewhere (Iran [74], Australia [40, 44, 49, 75], and Taiwan [47]).

There were 15 RCTs [36, 38, 40, 44, 45, 49, 51, 55, 67–72, 75], including 14 cluster RCTs [36, 38, 40, 44, 45, 49, 51, 55, 67–69, 71, 72, 75], and 15 CTs [37, 39, 41–43, 46–48, 52, 54, 60–62, 73, 74] including eight cluster CT studies [39, 41–43, 52, 54, 60, 61].

The duration of the interventions ranged from 6 weeks to 20 months. Ten studies [43–45, 48, 49, 54, 67, 69, 71, 72] lasted ≥6 months and seven studies [44, 45, 51, 54, 55, 62, 72] had a follow-up of 9 weeks to 18 months after the end of the intervention period.

The frequency of FMS intervention sessions given per week varied between once per week to daily. Five studies [41, 52, 67, 68, 72] offered an FMS intervention every day, 22 studies [36–39, 42–47, 49, 51, 54, 55, 60–62, 70, 71, 73–75] two to four times per week, one study [48] once a week and two studies [40, 69] did not specify the frequency. Fifteen studies [36, 38, 41, 43–45, 47, 49, 51, 55, 61, 67, 68, 72, 75] documented single intervention sessions lasting between 15 and 30 min, and 13 studies [37, 39, 42, 46, 48, 52, 54, 60, 62, 70, 71, 73, 74] between 30 to 65 min. Two studies [40, 69] did not provide any information for duration of a single session. All interventions were carried out in childcare or kindergarten settings (i.e., nursery center, early educational center, Head Start center). All interventions included either structured FMS sessions with additional unstructured time for physical activity in five trials [40, 42, 49, 52, 75] or only unstructured physical activity time but specifically devoted to improve FMS in two studies [43, 69]. In the structured FMS sessions, the intervention protocols consisted of an overall or specific training of FMS, including object control, locomotor and balance skill exercises, but also coordinative skills, rhythm with percussions and/or music, body awareness and perception, as well as games and creative movements, and improvisation skills. Unstructured physical activity time comprised defined free outdoor playtime and/or additional playground material to encourage physically active behavior and the development of FMS. Eight studies [36, 39, 45, 49, 51, 69, 71, 72] also focused on parental work (homework cards and physical activity home assignments for children with promotion of physical activity and FMS to parents) and nine studies set a focus on training sessions (workshops) for staff, nurses, and educators [36, 40, 43–45, 49, 67, 69, 75]. Four studies [36, 48, 52, 71] also taught the importance of healthy eating and nutrition to the children. To assess FMS (for a precise description of all tests see ESM Table S2), 16 studies [37–41, 46, 49, 51, 55, 60–62, 67, 70, 74, 75] used the TGMD—first or second edition, two studies [44, 68] used the BOT-2SF (Bruininks-Oseretsky Test of motor proficiency—Version 2 Short Form), two [48, 73] the MOT4-6 (Motorik Test for 4- to 6-year-old children), two [36, 47] the PDMS-2 (Peabody Development Motor Scale—2nd edition), and another eight studies [42, 43, 45, 52, 54, 69, 71, 72] used single items or other FMS test batteries.

### Risk of Bias

Overall, eight out of 30 studies (27%) [38, 40, 45, 51, 69, 71, 72, 75] were rated to be of high methodological quality (see ESM Table S4). A total of eleven studies [36, 40, 43–45, 49, 52, 55, 69, 71, 72] had >100 participants (of those, five studies [40, 45, 69, 71, 72] were of high quality). Just six studies applied intention-to-treat analyses [41, 46, 69, 71, 72, 75] but most studies measured the study groups at similar times. Insufficient information was provided to score the adequacy of the randomization procedure in nine studies [36, 38, 44, 49, 51, 67–70] (30%), and five studies [37, 38, 42, 47, 48] lacked information on allocation concealment or blinding of assessors at outcome assessment. Most studies reported detailed information regarding the intervention protocol for duration of training and training content (Table 1). However, the curriculum of the CON was not specified beyond usual care in 19 of the 30 studies.

### Effects of Interventions to Improve Fundamental Movement Skills

Findings from 26 out of 30 studies [36, 38–41, 43–46, 49, 51, 52, 54, 55, 60–62, 67–75] were aggregated and included in different meta-analytical calculations (ESM Table S5). For four studies [37, 42, 47, 48], results for meta-analytical calculations were not available. Results of those four studies lasting 6 weeks to 6 months included two studies [37, 47] that reported statistically significant differences for the LMS at post-intervention in favor of the INT, one study [48] found statistically significant differences for overall motor proficiency in favor of the INT, and one study [42] found no significant differences in FMS among groups.

**Fig. 2:**
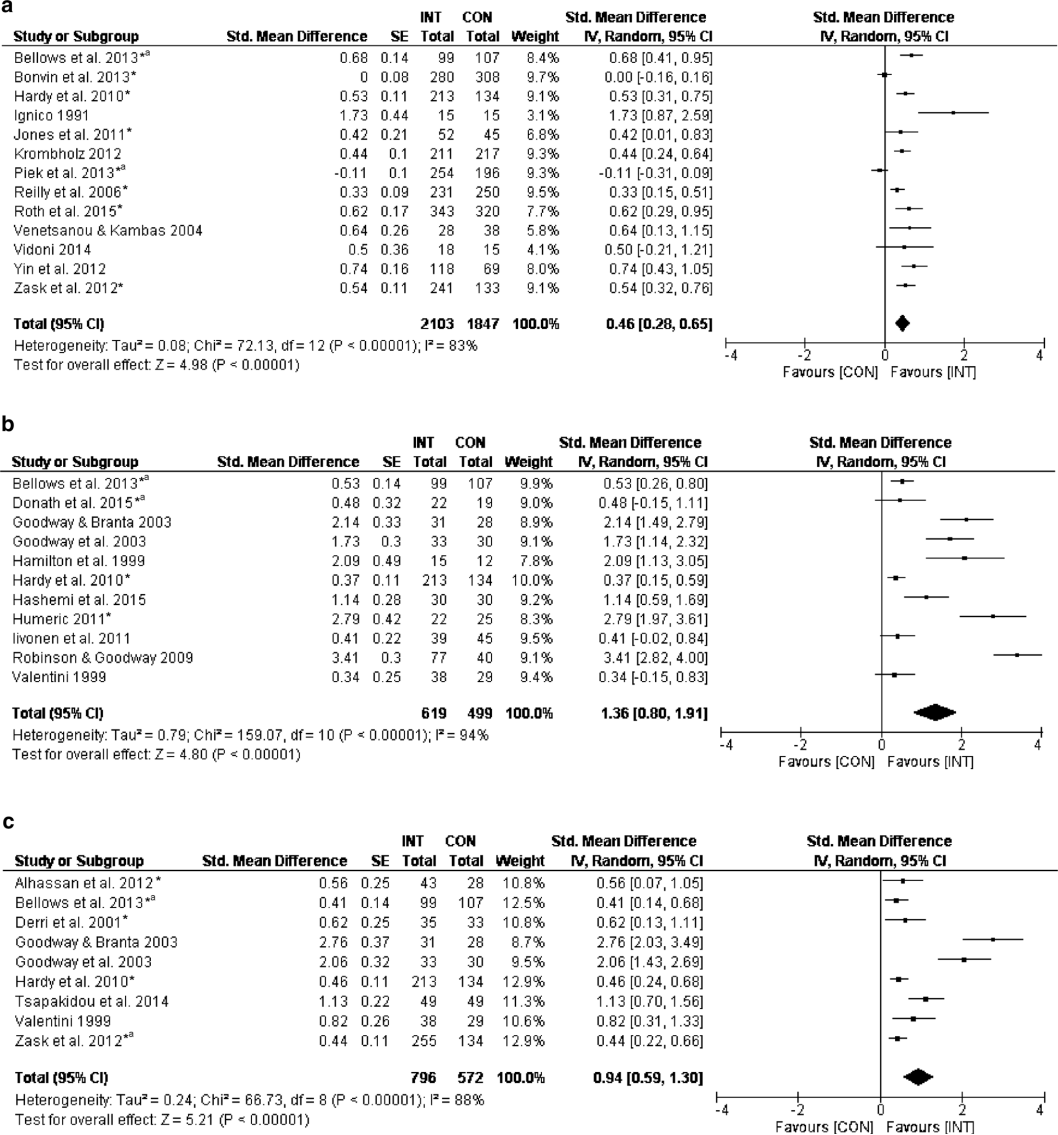
Effects of fundamental movement skills (FMS) interventions on **a** total FMS score (40-point scale, higher score is better), **b** object control subscale (OCS; 20-point scale, higher score is better), and **c** locomotor subscale (LMS; 20-point scale, higher score is better). *CI* confidence interval, *CON* control group, *INT* intervention group, *IV* inverse variance, *SE* standard error, *Std* standardized, *randomized controlled trial, ^a^additional information from author

Forest plots and summary results of the meta-analyses for total FMS, OCS, and LMS are described in Fig. 2 and Table 2. Thirteen [36, 40, 41, 43–45, 49, 52, 68, 69, 72, 73, 75] out of 26 studies which measured overall motor proficiency (total FMS score) showed small effects of the intervention programs on the INT compared with CON (weighted mean SMD_between_ = 0.46, 95% CI 0.28–0.65; *I* ^2^ = 83%, Fig. 2a). The subscale-specific analyses revealed large effects of intervention programs on the OCS in 11 [36, 38–40, 51, 54, 55, 60–62, 74] out of 26 studies (weighted mean SMD_between_ = 1.36, 95% CI 0.80–1.91; *I* ^2^ = 94%, Fig. 2b) and also large effects in nine studies [36, 40, 46, 49, 60–62, 67, 70] on the LMS (weighted mean SMD_between_ = 0.94, 95% CI 0.59–1.30; *I* ^2^ = 88%, Fig. 2c). Based on GRADE, there was very low certainty of evidence (Table 2) for effect sizes of the total FMS score and both subscale scores including, but not limited to, a high chance of a publication bias (ESM Fig. S1).

**Table 2:** GRADE evidence profiles: fundamental movement skills (FMS) enhancing intervention versus usual care

Quality assessment							No. of participants^f^		Absolute effect (95% CI)^f^	Quality	Importance
No. of studies	Study design	Risk of bias	Inconsistency	Indirectness	Imprecision	Other considerations	INT	CON			
*Overall FMS (follow-up: range 6* *weeks to 20* *months; assessed with or converted to TGMD-2; standard score from 2 to 40)*											
16	RCT and CT	Serious^a,b^	Serious^c^	Serious^d^	Not serious	Publication bias^e^	2103	1847	SMD 0.46 higher (0.28–0.65 higher)	Very low	Important
*OCS (follow-up: range 6* *weeks to 8* *months; assessed with or converted to TGMD-2; standard score from 1 to 20)*											
11	RCT and CT	Serious^a,b^	Serious^c^	Serious^d^	Not serious	Publication bias^e^	619	499	SMD 1.36 higher (0.80–1.91 higher)	Very low	Important
*LMS (follow-up: range 6* *weeks to 11* *months; assessed with or converted to TGMD-2; standard score from 1 to 20)*											
10	RCT and CT	Serious^a,b^	Serious^c^	Serious^d^	Not serious	Publication bias^e^	796	572	SMD 0.94 higher (0.59–1.30 higher)	Very low	Important

GRADE Working Group grade of evidence

High quality: We are very confident that the true effect lies close to that of the estimate of the effect

Moderate quality: We are moderately confident in the effect estimate: the true effect is likely to be close to the estimate of the effect, but there is a possibility that it is substantially different

Low quality: Our confidence in the effect is limited: the true effect may be substantially different from the estimate of the effect

Very low quality: We have very little confidence in the effect estimate: the true effect is likely to be substantially different from the estimate of the effect

*CI* confidence interval, *CON* control group, *CT* controlled trial, GRADE Grading of Recommendations Assessment, Development, and Evaluation System, *INT* intervention group, *LMS* Locomotor Subscale, *OCS* Object Control Subscale, *RCT* randomized controlled trial, *SMD* standardized mean difference

^a^Serious because of no clear randomization procedures described

^b^Serious because of selection bias (unclear or inadequate allocation concealment), detection bias (unclear blinding of data analysts), study integrity (unclear compliance with the intervention)

^c^Serious because of statistical heterogeneity (*I* ^2^ = 83–88%; *p* < 0.0001)

^d^Serious because of important differences in implementation across settings

^e^Serious because publication bias possible

^f^3 and 1 studies for overall FMS and LMS scores, respectively, could not be included in meta-analyses

ESM Figs. S2–S4 illustrate forest plots of the intervention effects for single motor skill items integrated in the TGMD-2 scores, and other skills like the standing long jump and balance. Intervention effects were statistically significant in favor of INT for all single items, with effect sizes ranging from low to moderate (0.19–0.83). There was only a small number (i.e., 3–7) of studies in each meta-analysis and a high heterogeneity with *I* ^2^ ranging from 73 to 90%, except for the standing long jump that showed an *I* ^2^ = 0%. There was no clear picture regarding characteristics of the interventions (frequency, duration), target population (disadvantaged children, age), or setting (childcare, kindergarten) that explained why the effectiveness in total FMS and subscales varied considerably.

### Subgroup and Exploratory Analyses

#### Subgroup Analyses

**Fig. 3:**
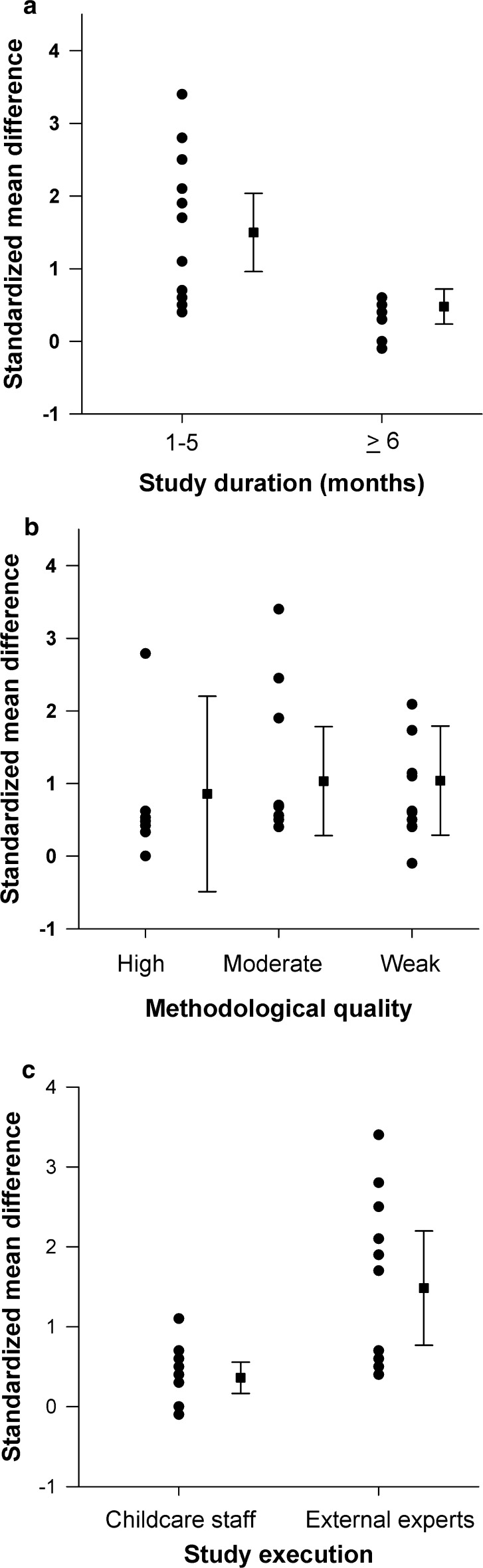
Effect sizes of fundamental movement skill (FMS) interventions according to **a** duration, **b** methodological quality, and **c** study execution of included studies. *Filled circles* illustrate standardized mean differences (SMD_between_) between intervention and control group for single studies. The *filled squares* represent weighted mean SMD_between_ with 95% confidence intervals (CI) of the studies combined. The figures show **a** statistically significant higher effect sizes on overall FMS in favor of studies with shorter duration (SMD_between_ = 1.23, 95% CI 0.86–1.61) compared with studies with longer duration (SMD_between_ = 0.32, 95% CI 0.12–0.52); **b** no statistically significant differences in effect sizes on overall FMS for studies of ‘high’ methodological quality (SMD_between_ = 0.59, 95% CI 0.26–0.93) compared with studies with ‘moderate’ (SMD_between_ = 1.31, 95% CI 0.74–1.88) and ‘weak’ (SMD_between_ = 0.76, 95% CI 0.40–1.11) methodological quality; and **c** statistically significant higher effect sizes on overall FMS in favor of studies with external experts (SMD_between_ = 1.54, 95% CI 0.93–2.15) compared with childcare staff (SMD_between_ = 0.41, 95% CI 0.23–0.59)

Figure 3a displays the overall dose–response relationship according to the duration of the interventions. The 17 trials [36, 38–41, 46, 51, 52, 55, 60–62, 68, 70, 73–75] with a shorter duration (4 weeks to 5 months) showed significantly higher effect sizes on overall FMS compared with those eight studies [43–45, 49, 54, 67, 69, 72] with longer duration (≥6 months) (weighted mean SMD_between_ = 1.43, 95% CI 0.49–2.38). Four studies [37, 42, 47, 48] did not report their results and, for one study [71], data were available only for single items. Figure 3b presents the intervention effects for 25 trials [36, 38–41, 43–46, 49, 51, 52, 54, 55, 60–62, 67–70, 72–75] according to methodological quality. Eight studies [36, 43, 49, 52, 55, 60, 61, 67] with ‘moderate’ (weighted mean SMD_between_ = 1.00, 95% CI −0.09 to 2.10) and ten studies [39, 41, 44, 46, 54, 62, 68, 70, 73, 74] with ‘weak’ (weighted mean SMD_between_ = 0.27, 95% CI −0.64 to 1.18) methodological quality showed no statistically significant differences in effect sizes on overall FMS compared with the seven studies [38, 40, 45, 51, 69, 72, 75] of ‘high’ methodological quality. For total FMS we compared studies that used the TGMD-2 test versus others that used different tests. There was no significant difference in effect sizes between the four studies [40, 41, 49, 75] that used the TGMD-2 and the nine studies [36, 43–45, 52, 68, 69, 72, 73] that used another test (weighted mean SMD_between_ = 0.72, 95% CI −0.50 to 1.94).

#### Exploratory Analyses

Nine [41, 44–46, 49, 51, 54, 61, 62] out of 30 studies in this systematic review looked at some aspects of gender differences but results were too heterogeneous to run meta-analyses. Effects in girls compared with boys for total FMS were larger in three [41, 45, 49] and smaller in one study [44]. For locomotor skills, no difference in effect sizes were found between the sexes in three studies [46, 61, 62]. However, consistently larger effects were found for object control skills in boys compared with girls in four studies [51, 54, 61, 62]. There was no clear picture regarding characteristics of the interventions (frequency, duration), target population (disadvantaged children, age) or setting (childcare, kindergarten) that explained gender differences in results. Four studies [39, 60–62] included disadvantaged children or children that were at risk of delay in FMS competence due to socioeconomic or biological factors. Three of these studies [39, 60, 61] showed particularly large effect sizes (SMD_between_) for LMS and OCS (2.06–2.76).

Figure 3c shows the intervention effects according to the persons who implemented the FMS intervention in childcares or kindergartens. The 11 studies [38, 39, 41, 51, 52, 55, 60–62, 67, 73] in which external experts implemented the intervention programs compared with the 12 studies [36, 40, 43–46, 49, 54, 68, 69, 72, 75] in which childcare or kindergarten teachers were responsible for implementation showed statistically significant higher effect sizes on overall FMS (weighted mean SMD_between_ = 1.46, 95% CI 0.52–2.40). For five studies [37, 42, 47, 48, 71], results were not available due to missing SMD, SD, and/or SE or due to the reporting of only single items. Whether studies were more or less effective was not differentiated by either the setting where FMS interventions took place (kindergarten versus childcare), the use of a theoretical framework on which the intervention was based (yes versus no), or the additional involvement of parents in FMS intervention programs (yes versus no) (data not shown). In addition, we were unable to tease out the most effective intervention approach based on pedagogic concept, the volume or the content of the interventions to improve and develop FMS.

## Discussion

Our systematic review and meta-analyses revealed beneficial effects on overall motor skill proficiency (total FMS score), as well as on object control and locomotor skills in children aged 2–6 years with small-to-large effect sizes following FMS intervention programs conducted in childcare or kindergarten settings. Further, studies of shorter (<6 months) compared with longer duration (≥6 months) and the integration of external experts rather than implementation of the programs by the usual childcare/kindergarten teachers resulted in higher effect sizes, while the methodological quality of the studies did not play a role. Importantly, due to the low certainty of evidence based on GRADE, findings of this systematic review and meta-analysis have to be interpreted with care. Even though most studies conducted in childcare and kindergarten proved to be effective, we have to acknowledge that the effect estimates and the true effect may likely be substantially different from the current effect estimates as reported in this review. This finding should by no means be interpreted as that FMS interventions in young children should not be done as there is insufficient evidence, but rather, it should be taken as a key message that more high-quality research is needed in the field of FMS interventions in early childhood [76]. A higher quality of studies would imply high-standard randomization procedures, the careful selection of control groups to prevent cross-contamination [77, 78], the integration of appropriate power analyses to calculate sample sizes needed for group or sub-group analyses (e.g., for gender), and the blinding of assessors for important outcomes such as FMS [79, 80]. Further, it seems imperative and timely to carefully select and standardize test batteries for FMS assessment [81], to use adequate statistical methods including appropriate baseline comparisons as well as the control for important confounders and clusters [82], to assess intervention fidelity [83], and finally to integrate long-term follow-up [84].

### Interpretation of Overall, Subgroup, and Exploratory Analyses

Despite our comprehensive search in seven databases from the year of inception up to August 2015, only 30 studies fulfilled our eligibility criteria, 15 of which [37, 39, 41–43, 46–48, 52, 54, 60–62, 73, 74] were CTs rather than RCTs. There was, however, no major difference in findings and effect sizes between CTs or RCTs (data not shown). Contrary to physiological considerations of a dose-response principle with the expectation that longer interventions would lead to higher effect sizes, we found that longer interventions showed smaller effect sizes (Fig. 3a). This trend was also documented in other reviews [31] and suggests that a loss of compliance and motivation may have occurred with activities provided during FMS interventions becoming monotonous and leading children and caregivers to lose interest over time [25]. Alternatively, there may have been insufficient adaption of the programs, which need training progression over time to keep up a stimulus [85, 86].

The methodological quality of the studies was not proportional to the effect sizes of the intervention on FMS (Fig. 3b), suggesting that an overestimation of training on FMS in preschoolers did not occur. It is also reassuring that the overall picture of beneficial effects of interventions on overall FMS, OCS, and LMS was consistent and in accordance with other reviews focusing on children with developmental delays [27, 28] or on children with an older age range [25, 26]. Even in the single test items, findings revealed medium (jumping, throwing, catching, kicking) or at least small (running, hopping, standing long jump, balance) effect sizes. Yet, based on GRADE (Table 2), where we assessed the magnitude of effects and the overall quality of evidence and found that the estimates of FMS interventions in young children are trustworthy, we have little confidence in the effect estimates and it is therefore very probable that the true effect is likely substantially smaller or larger than the effect estimate. Of the five relevant factors that can lower the quality of evidence, four factors showed serious limitations. These included the failure of describing the detailed study design and execution or risk of bias (e.g., no clear description of randomization procedures), the finding of inconsistency or heterogeneity of effects (e.g., statistical heterogeneity of effects with *I* ^2^ > 80% all outcomes), indirectness or applicability (e.g., important differences in implementation across settings), and a possible publication bias (ESM Fig. S1). Smaller estimates of effects of FMS interventions may, for instance, be found if assessors of FMS are blinded for group assignments [79, 80], while larger effects may be found if fidelity regarding the implementation of the intervention is assessed [83], or by the selection of proper control groups without cross-contamination [77, 78].

Although some argue that long-term follow-ups are most relevant when studies show short-term effects, follow-ups should be contingent on the methodological quality of the original trial, irrespective of effect [76]. In this review, only seven [44, 45, 51, 54, 55, 62, 72] of 30 studies included longer-term follow-ups. Of those, three studies [51, 55, 72] provided evidence of sustained beneficial effects on FMS 8–12 weeks off intervention (SMD_between_ = 1.80, 95% CI 1.03–2.57; SMD_between_ = 0.59, 95% CI 0.17–1.01; SMD_between_ = 2.67, 95% CI 2.15–3.19), while four studies [44, 45, 54, 62] with follow-up from 3–12 months off intervention did not find lasting effects. This finding supports the opinion of experts in the field that FMS have to be taught, practiced, and reinforced repeatedly as they do not seem to develop and be maintained naturally [26, 31, 32]. However, it may be a challenge to find feasible and effective strategies that lead to a sustained FMS proficiency in view of the fading effects with longer-term interventions and the obvious need for experienced teachers.

In order to help us better understand which intervention strategies may or may not work, why, and for whom, we tried to tease out interventions that were more effective than others by stratifying for target groups, the setting, and characteristics of the interventions. Although trials were only included if they examined typically developing young children, four studies [39, 60–62] included disadvantaged children or children that were at risk of delay in FMS competence due to socioeconomic or biological factors. Three studies [39, 60, 61] showed particularly large effect sizes (SMD_between_) for LMS and OCS (2.06–2.76), possibly because these children may have had greater potential to improve FMS competence [31]. On the other hand, interventions targeting a completely healthy population of young children may have the problem of attaining a ceiling effect in FMS proficiency. This could be the case when FMS interventions use an FMS outcome test that is mainly built to differentiate typically developing children from those with a motor deficiency rather than having the potential to differentiate skills within a healthy population [8]. We do not think that this phenomenon has occurred, as in our review, effect sizes for total FMS among those studies that started with mean values below the median at baseline were not different from those that started with above-median values (SMD_between_ = 1.01, 95% CI −0.11 to 2.29). A ceiling effect for FMS intervention results may also be more likely when the age of the target group children is close to the upper limit of the validated age range that is covered by the respective test battery [81]. This was not the case in most studies in this review. Firstly, they used scaled scores or percentiles for age categories based on half-yearly or yearly steps to adjust for age and maturational effects; and secondly, they used predominantly the TGMD(-2), which covers ages up to 10 years. Nevertheless, several tests that were also used in the included studies have an upper age limit of 6 years (see ESM Table S2), where a ceiling effect might have played a role. As studies usually report mean ages and SDs, the ceiling effect is difficult to assess, but should indeed be considered in future studies.

Although clear gender differences for FMS exist [15, 87, 88], be it related to differences in physical activity behavior [89] or cultural norms [8] that may foster enhanced FMS in boys (e.g., kicking) or girls (e.g., balancing), the reach and responsiveness of girls and boys in interventions targeting FMS may be different as well. Only a few studies in this review scrutinized gender differences. They reported unequivocal results for total FMS (one study [44] with higher effect sizes in boys and three studies [41, 45, 49] with higher effect sizes in girls), consistently better results for object control skills favoring boys (four studies [51, 54, 61, 62]), but no difference in effects for locomotor skills (three studies [46, 61, 62]). Although recent primary research focusing on FMS indicated that gender differences in FMS existed in favor of the boys [90–93], FMS was a predictor of physical activity and fitness in adolescence in both sexes [6, 94]. The few gender-differentiated results in our systematic review did not allow for conclusions to be drawn on whether girls or boys profited more from FMS interventions or whether there is a need for and value to be gained from targeting. So far, both sexes seem to profit from FMS interventions. It may be that boys profit more from interventions targeting object control skills, as consistently stronger effects in favor of boys were found in our review [51, 54, 61, 62]. Perceived competence, whether preceded [95] or as a consequence of actual (motor) competence [96], may have played a role in their motivation to improve object control skills [97, 98]. However, evidence of a gender difference in the association between actual and perceived FMS in young children is lacking [99]. As Barnett et al. [90] suggested, boys may simply obtain more encouragement, positive reinforcement, and stimulation for activities involving object control skills.

Future consideration should therefore be given to the need for a universal or gender-targeted approach, the acceptability and effectiveness of different approaches available for targeting, and the potential positive and negative consequences of either [76].

While the setting (kindergarten versus childcare) did not play a role in effectiveness, effects were stronger when the intervention was provided by an external expert in the field of FMS rather than the usual childcare or kindergarten teachers (Fig. 3c). The integration of experts to build up proper FMS programs and educate childcare and kindergarten teams how to teach FMS [100] seems evident [55]. These experts bring the combined expertise of knowledge about the development and training of FMS and the pedagogic skills needed to foster actual but also perceived FMS [100]. They may also be more skilled at providing the magic intervention ingredient of fun that is identified as a critical component of interventions [101, 102] and that may lead to sustained enjoyment [103, 104] and create a motivational climate for teachers and the children [62]. Promising concepts have been used by the integrated studies attempting to integrate these fundamental psychological and pedagogic principles, including programs that specifically focused on a mastery climate [55], or integrated music and dance [37, 46, 62, 68, 70]. Whatever the concept, an intervention delivering on sustained fun is likely to engage children as well as teachers and promote ongoing involvement, while being enjoyable to deliver [76].

### Strengths and Limitations

Our review has several strengths. We reviewed all intervention studies aimed at increasing motor skills in young children by including a larger range of literature databases than other reviews [26, 31, 32]. The focus was on typically developing young children attending childcare or kindergarten in contrast to mainly school-aged individuals [25, 26] and did not include children with existing motor handicaps or with developmental delays [27, 28]. Teams of reviewers worked both independently and in pairs to select eligible studies, assess risk of bias and extract data. Furthermore, we used the GRADE approach to rate our certainty in the evidence and presented findings with the GRADE evidence profiles. Our results are limited by shortcomings of many of the studies that were eligible for our review and led to our ratings of very low certainty for the intervention effects. Reasons for downgrading included limitations in the study design such as CTs or RCTs with unclear randomization procedures and lack of information regarding allocation concealment, and lack of blinding of outcome assessors and data analysts. Moreover, there was a huge variation in intervention content, duration, and intensity, and often an unknown intervention integrity that did not lead to any sort of dose-response in the outcomes [65]. In addition, there was a large heterogeneity of results. This heterogeneity of results may be explained at least in part by the substantial variation in intervention load and strategies, by the use of a wide range of motor test batteries to measure motor skills [105], or by a high chance of a publication bias. The latter is shown in the consistently asymmetrical funnel plots for the overall FMS and the subscales [106] (ESM Fig. S1) and verified by the Egger’s test [107] (data not shown). The activities in the control group were poorly defined in 19 out of 30 studies, providing room for bias [77, 78]. Further limitations were the exclusion of studies written in languages other than English or German, the skipping of the forward tracking of studies (e.g., looking at studies that cite the included articles), and the conversion of the motor skill test results to the most commonly used test battery among the eligible studies of this review as suggested by GRADE [66]. These applied motor skill test batteries may appear to measure similar constructs and show high correlations in change scores; however, responsiveness of instruments may differ substantially and lead to important between-study heterogeneity [108]. Nevertheless, in our review effect sizes for total FMS were similar in studies that used the reference test (TGMD[-2]) versus those that used another test, suggesting the different responsiveness was not a major problem. Moreover, the use of process-oriented FMS tests that measure how (well) a movement skill is measured or product-oriented FMS assessment batteries in which quantity aspects (e.g., time or distance) are measured provide diverging information [81]. Although the two means of assessment are reasonably related, they also show substantial variation of correlations that may have affected the pooled results in meta-analyses [8, 81].

### Implications for Clinical Practice

From a very young age, proficiency in FMS is related to relevant aspects of health including higher physical activity and physical fitness, reduced obesity, and enhanced social and cognitive skills [11, 109]. Developing motor skills enables the young child to interact with the social and physical environment. As children grow, motor skills are crucial to engage in a large variety of movements and play activities, starting with simple running or throwing a ball to complex physical interactions with peers in the playground or during (organized) sports. Moreover, mutual interactions between motor and cognitive performance and executive functions take place [110, 111] and motor control is used to guide the way in which the surroundings are perceived and processed through ongoing interactions between brain, body, and environment [112]. Thus, improving actual motor skill development, but also perceived motor competence may provide enhanced opportunities for the development of a variety of perceptual, social, and cognitive skills, and may further be influenced in turn by these abilities in iterative interactive cycles [9, 98, 113, 114]. Given these clinically relevant and plausible benefits, improving actual and perceived motor skills should be a priority public health strategy to stimulate physical activity in youth, ideally implemented at the childcare or kindergarten level where a large number of young children can be reached very early [9, 31, 32] and without stigmatization of those that need it most.

Based on this and previous reviews [26, 31, 32], all aspects of FMS should and can be taught in childcare, kindergarten, or similar settings, including object control skills, locomotor skills, balance, or more complex FMS tasks (see Fig. 2 and ESM Figs. S2–4), preferably by the integration of an expert teacher [55] and by intervening over time [26, 31, 32]. Careful emphasis should be placed on maintaining attractive and potent intervention programs for children and teachers as effects may fade with time due to a loss of motivation or insufficient physical stimulus. To progress the field, more theory-driven research [9] needs to be done to tease out the most effective intervention components (length and intensity of sessions, timing, duration, content, context such as with or without music, the integration of dance items), as well as possible effect modifications by age [115], gender [116], obesity [15], physical activity [10], perceived motor competence [97, 117], physical fitness [14], characteristics of the setting [69], and teachers [15].

Scientifically, the best strategy to improve FMS in young children has yet to be determined in future studies that will hopefully address current limitations. The conduct and publication of well-designed evaluations of well-defined interventions using the same standardized assessment tool for young children, preferably combining process- and product-oriented FMS test items [81], with international reference values allowing direct comparison (also of intervention effects) worldwide is crucial to advance the field of FMS promotion in children and help us better understand which intervention strategies may or may not work, why, and for whom [76]. Consequently, this may then lead to realistic and clinically sound implementation strategies to foster FMS proficiency starting at an early age.

## Conclusion

This review indicates positive effects of childcare- or kindergarten-based interventions on FMS proficiency in young children. Yet, the evidence base is low and we have little confidence in the effect estimate. As the true effect is likely to be substantially different from the reported estimate of the effect, results must be considered with care. Nevertheless, FMS-enhancing programs may have an important role in children attaining motor skill proficiency as the basis for a physically active lifestyle [6] and to profit from a variety of physiological, social, and cognitive health benefits [11, 118]. Future high-quality research is needed to establish certainty in effectiveness of FMS training in young children by searching for optimal programs, looking at dose-response relations and long-term sustainability.

Additional references can be found in the ESM [8, 33, 36, 37, 88, 119–132].

## Electronic supplementary material

Below is the link to the electronic supplementary material.

**Electronic Supplementary Material Fig. S1** Funnel plots for (a) total fundamental movement skills (FMS) score, (b) object control subscale (OCS), and (c) locomotor subscale (LMS) of included studies with *lines* representing the pooled estimates and *circles* representing point estimates of the studies compatible with probable publication bias (confirmed by Egger’s regression test). (PDF 172 kb)

**Electronic Supplementary Material Fig. S2** Effects of fundamental movement skill (FMS) interventions (INT) versus control (CON; with usual childcare) on measures of single items of the object control subscale (OCS): (a) catching, (b) kicking, and (c) throwing. *CI* confidence interval, *CON* control group, *INT* intervention group, *IV* inverse variance, *SE* standard error, *Std* standardized, * RCT, ^a^ Additional information from author (PDF 190 kb)

**Electronic Supplementary Material Fig. S3** Effects of fundamental movement skills (FMS) interventions (INT) versus control (CON; with usual childcare) on measures of single items of the locomotor subscale (LMS): (a) running, (b) jumping, (c) hopping, and (d) standing long jump. *CI* confidence interval, *CON* control group, *INT* intervention group, *IV* inverse variance, *SE* standard error, *Std* standardized, * RCT, ^a^ Additional information from author (PDF 198 kb)

**Electronic Supplementary Material Fig. S4** Effects of fundamental movement skills (FMS) interventions (INT) versus control (CON; with usual childcare) on measures of balance. *CI* confidence interval, *CON* control group, *INT* intervention group, *IV* inverse variance, *SE* standard error, *Std* standardized, * RCT, ^a^ Additional information from author (PDF 41 kb)

Electronic Supplementary Material Table S1 (DOCX 16 kb)

Electronic Supplementary Material Table S2 (DOCX 37 kb)

Electronic Supplementary Material Table S3 (DOCX 21 kb)

Electronic Supplementary Material Table S4 (DOCX 35 kb)

Electronic Supplementary Material Table S5 (DOCX 36 kb)

## Abbreviations

CI: Confidence interval
CON: Control group
CT: Controlled trial
EPHPP: Effective Public Health Practice Project—Quality Assessment Tool for Quantitative Studies
FMS: Fundamental movement skills
GRADE: Grading of Recommendations Assessment, Development, and Evaluation System
INT: Intervention group
LMS: Locomotor subscale
OCS: Object control subscale
PRISMA: Preferred Reporting Items for Systematic Reviews and Meta-Analyses
RCT: Randomized controlled trial
SD: Standard deviation
SE: Standard error
SMD: Standardized mean difference
WoS: Web of Science
