# Supplementary figures and images for: Interventions to Promote Fundamental Movement Skills in Childcare and Kindergarten: A Systematic Review and Meta-Analysis
Source: Sports Med. 2017 Apr 6;47(10):2045–68. doi: 10.1007/s40279-017-0723-1 (PMC5603621; doi:10.1007/s40279-017-0723-1)

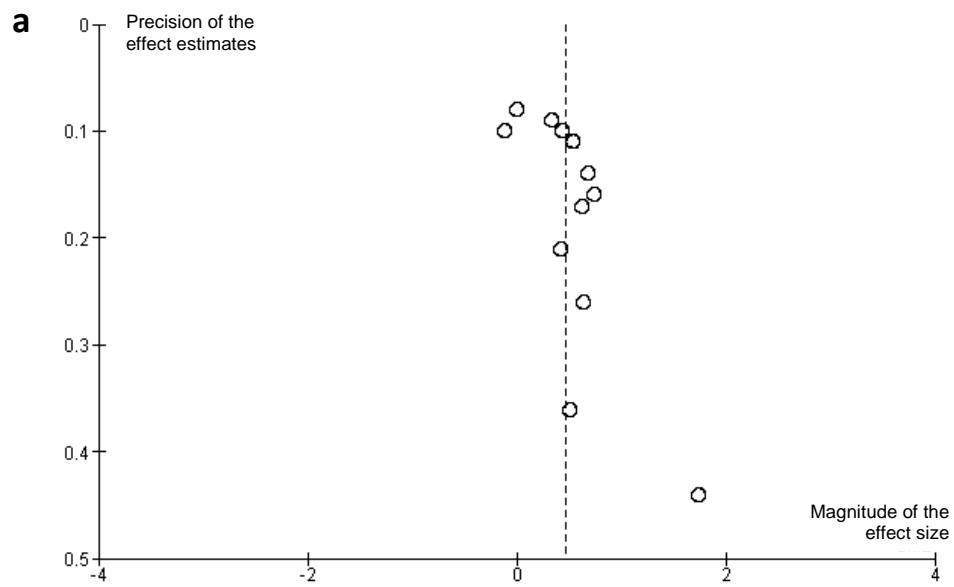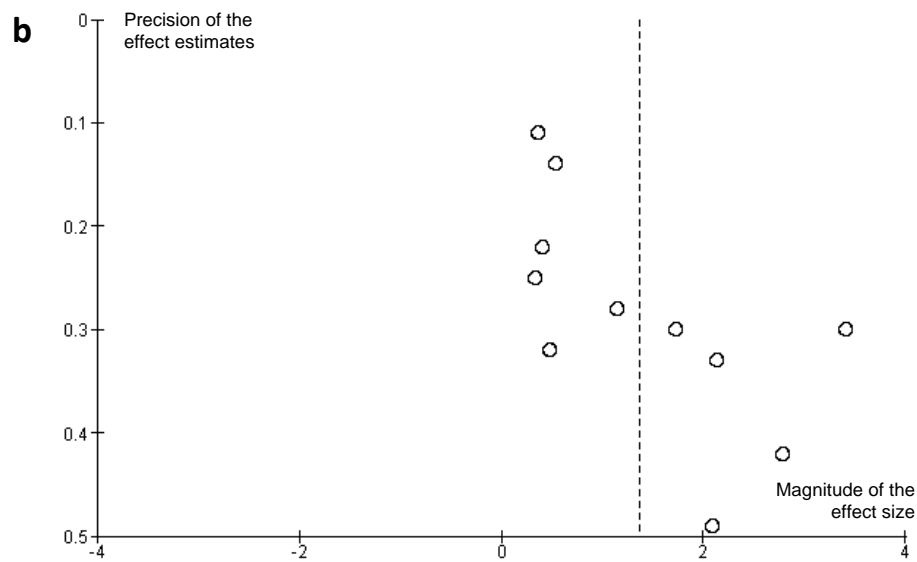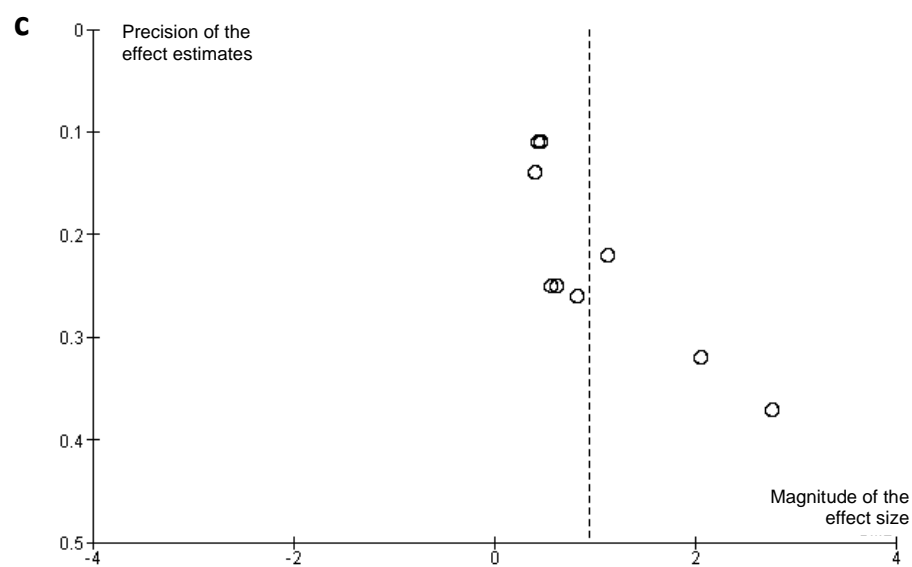

Supplement: Supplementary file 1 — Electronic Supplementary Material Fig. S1 Funnel plots for (a) total fundamental movement skills (FMS) score, (b) object control subscale (OCS), and (c) locomotor subscale (LMS) of included studies with lines representing the pooled estimates and circles representing point estimates of the studies compatible with probable publication bias (confirmed by Egger’s regression test). (PDF 172 kb) [file 40279_2017_723_MOESM1_ESM.pdf]

**a**

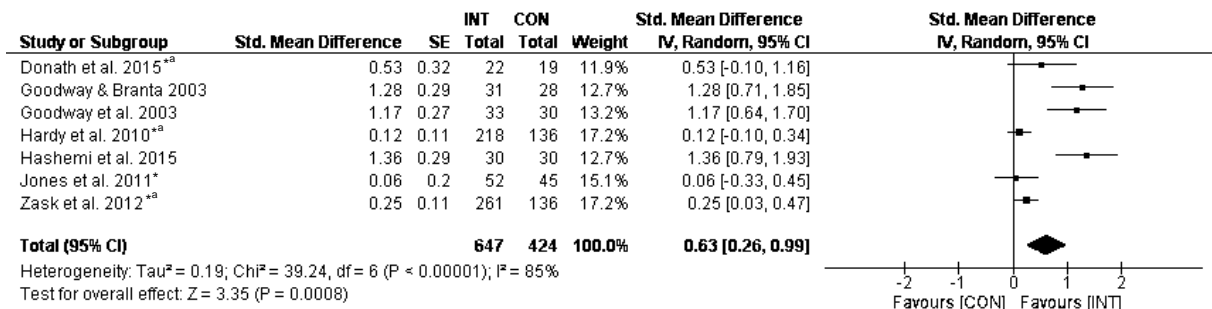

**b**

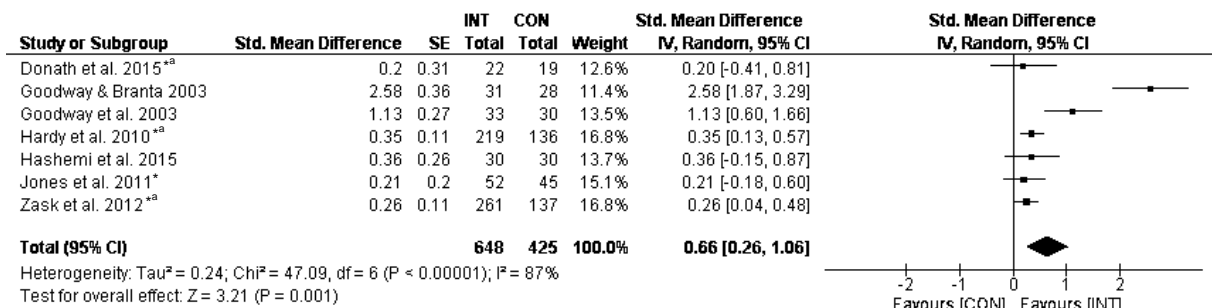

**c**

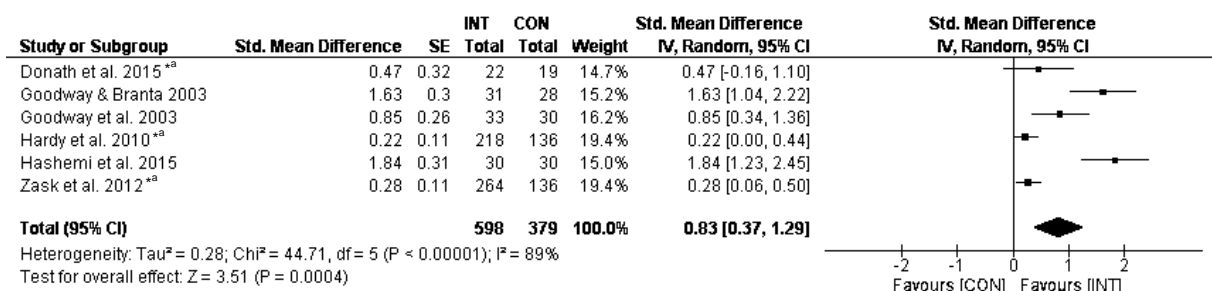

Supplement: Supplementary file 2 — Electronic Supplementary Material Fig. S2 Effects of fundamental movement skill (FMS) interventions (INT) versus control (CON; with usual childcare) on measures of single items of the object control subscale (OCS): (a) catching, (b) kicking, and (c) throwing. CI confidence interval, CON control group, INT intervention group, IV inverse variance, SE standard error, Std standardized, * RCT, a Additional information from author (PDF 190 kb) [file 40279_2017_723_MOESM2_ESM.pdf]

**a**

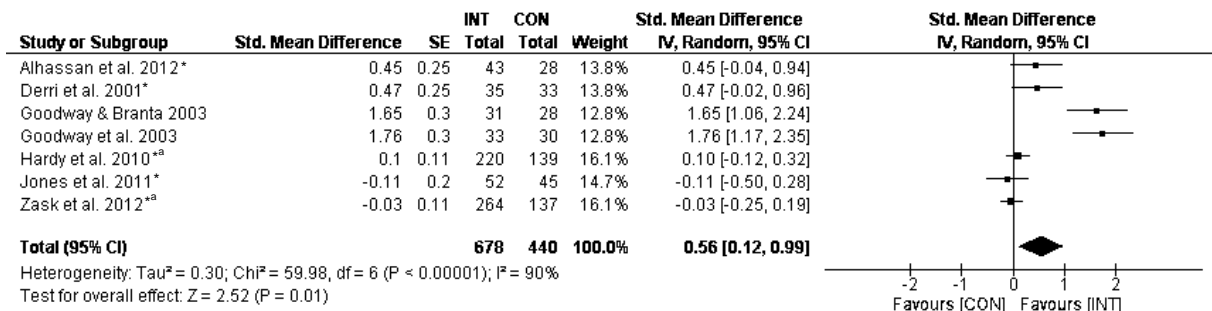

**b**

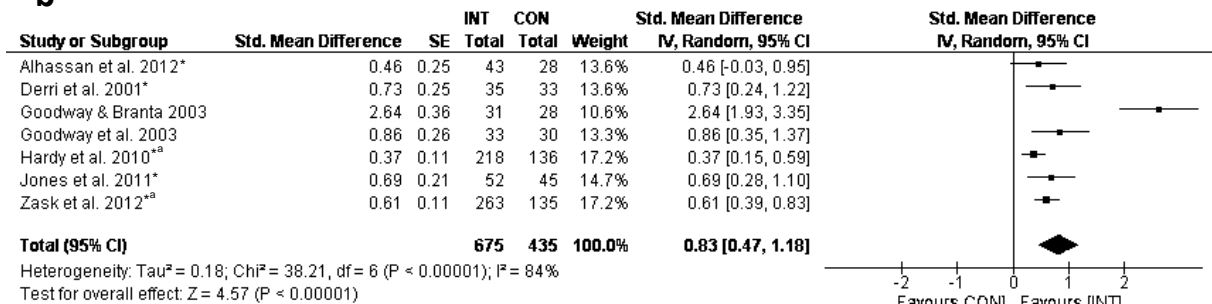

**c**

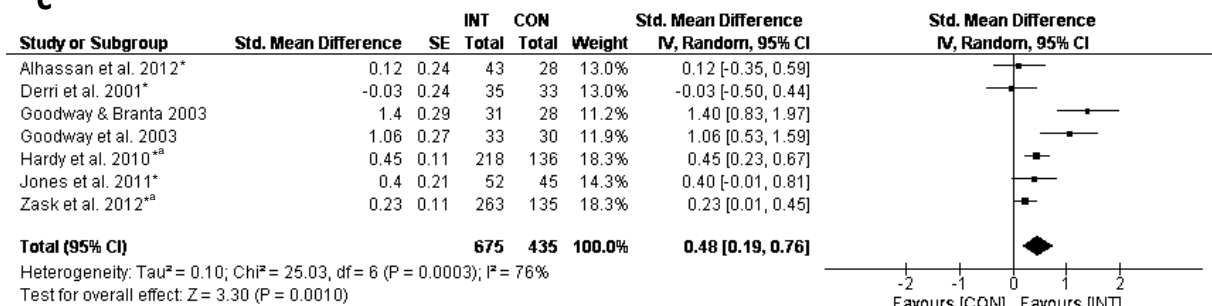

**d**

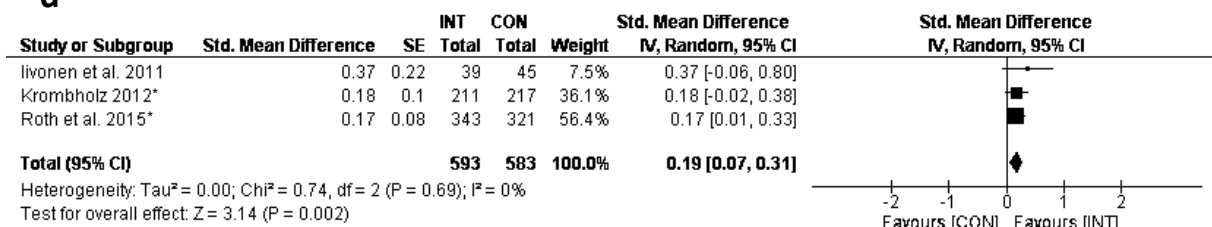

Supplement: Supplementary file 3 — Electronic Supplementary Material Fig. S3 Effects of fundamental movement skills (FMS) interventions (INT) versus control (CON; with usual childcare) on measures of single items of the locomotor subscale (LMS): (a) running, (b) jumping, (c) hopping, and (d) standing long jump. CI confidence interval, CON control group, INT intervention group, IV inverse variance, SE standard error, Std standardized, * RCT, a Additional information from author (PDF 198 kb) [file 40279_2017_723_MOESM3_ESM.pdf]

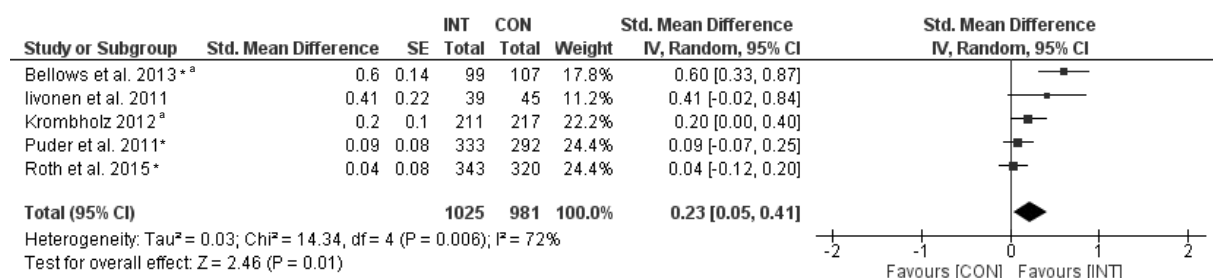

Supplement: Supplementary file 4 — Electronic Supplementary Material Fig. S4 Effects of fundamental movement skills (FMS) interventions (INT) versus control (CON; with usual childcare) on measures of balance. CI confidence interval, CON control group, INT intervention group, IV inverse variance, SE standard error, Std standardized, * RCT, a Additional information from author (PDF 41 kb) [file 40279_2017_723_MOESM4_ESM.pdf]
