# Supplementary material for: Interventions to Promote Fundamental Movement Skills in Childcare and Kindergarten: A Systematic Review and Meta-Analysis
Source: Sports Med. 2017 Apr 6;47(10):2045–68. doi: 10.1007/s40279-017-0723-1 (PMC5603621; doi:10.1007/s40279-017-0723-1)
Supplement: Supplementary file 5 — Electronic Supplementary Material Table S1 (DOCX 16 kb) [file 40279_2017_723_MOESM5_ESM.docx]

| **Electronic Supplementary Material Table S1.** Search strategy exemplary for MEDLINE | |
| --- | --- |
| S1 | ((MH "Child, Preschool") OR TI ( child OR children OR infant OR infants OR preschool OR kindergarden OR kindergarten OR pediatric OR paediatric OR paediatry OR boy OR boys OR boyhood OR girl OR girls OR girlhood OR grandchild ) OR AB ( child OR children OR infant OR infants OR preschool OR kindergarden OR kindergarte OR pediatric OR paediatric OR paediatry OR boy OR boys OR boyhood OR girl OR girls OR girlhood OR grandchild )) |
| S2 | (MH "Motor Skills") OR (MH "Motor Activity+") OR (MH "Exercise Movement Techniques+") OR (MH "Physical Fitness") OR (MH "Running+") OR (MH "Sports+") OR TI ( ((motor OR movement) N3 (skill* OR development OR ability)) ) OR AB ( ((motor OR movement) N3 (skill* OR development OR ability)) ) OR TI ( ((sport OR exercise OR “physical activity” OR walking OR coordination OR fitness OR play OR running OR galloping OR hopping OR leaping OR jumping OR striking OR dribbling OR catching OR kicking OR agility OR balance OR speed OR power OR "reaction time") N10 (program OR programme OR intervention)) ) OR AB ( ((sport OR exercise OR "physical activity” OR walking OR coordination OR fitness OR play OR running OR galloping OR hopping OR leaping OR jumping OR striking OR dribbling OR catching OR kicking OR agility OR balance OR speed OR power OR "reaction time") N10 (program OR programme OR intervention)) ) |
| S3 | S1 AND S2 |
| S4 | ((MH "Chronic Disease") OR (MH "Disabled Children") OR (MH "Cerebral Palsy") OR (MH "Neoplasms+") OR (MH "Cardiovascular Diseases+") OR (MH "Endocrine System Diseases+") OR (MH "Hemic and Lymphatic Diseases+") OR (MH "Immune System Diseases+") OR (MH "Musculoskeletal Diseases+") OR (MH "Neoplasms+") OR (MH "Nervous System Diseases+") OR (MH "Respiratory Tract Diseases+") OR (MH "Virus Diseases+") OR (MH "Wounds and Injuries+") OR TX (Disability OR disabilities OR handicapped OR ill OR disorder OR preterm OR "cerebral palsy" OR "prenatal alcohol exposure" OR medulloblastoma OR cancer OR tumour OR tumor OR tumors OR tumours) OR TX (chronic* N3 disease*)) |
| S5 | ((MH "Population Surveillance+") OR TI ( health* OR (normal* N3 (developed OR child*) OR (general N3 population) ) OR AB ( health* OR (normal* N3 (developed OR child*) OR (general N3 population) ) |
| S6 | S3 NOT (S4 NOT S5) |
| S7 | (MH "Randomized Controlled Trials as Topic+") OR (MH "Randomized Controlled Trial+") OR TI (control* OR effect* OR assess* OR evaluat* OR infuence OR impact OR outcome OR random*) N9 (intervention* OR study OR studies OR program OR programme OR trial* OR design) OR TI ( "controlled trial" OR "controlled study" OR "controlled intervention" OR "control group" OR "control groups" OR "intervention group") OR AB ( "controlled trial" OR "controlled study" OR "controlled intervention" OR "control group" OR "control groups" OR "intervention group")) |
| S8 | S6 AND S7 |
