# Supplementary material for: Interventions to Promote Fundamental Movement Skills in Childcare and Kindergarten: A Systematic Review and Meta-Analysis
Source: Sports Med. 2017 Apr 6;47(10):2045–68. doi: 10.1007/s40279-017-0723-1 (PMC5603621; doi:10.1007/s40279-017-0723-1)
Supplement: Supplementary file 6 — Electronic Supplementary Material Table S2 (DOCX 37 kb) [file 40279_2017_723_MOESM6_ESM.docx]

| **Electronic Supplementary Material Table S2.** Assessment Tools of Fundamental Movement Skills (FMS) Used in Included Studies | | | | | | | | | |
| --- | --- | --- | --- | --- | --- | --- | --- | --- | --- |
| **Assessment^a^** | **Name of Test** | **Age Range (years)** | **Items^b^/ Age Bands** | **Object Control Skills** | **Locomotor Skills** | **Balance Skills** | **Reliability Aspects** | **Validity Aspects** | **Studies^c^** |
| APM Inventory | **Alle kouluikäisten lasten PsykoMotoriset taidot (1995)** | 1 to 7 | N/A | **Single Items:**  - Throwing and catching combination  - Throwing at a target  - Kicking ball at target | **Single Items:**  - Running (10 m) (Time in sec)  - Standing broad jump (in cm) | **Single Items:**  - Static balance – standing on the right and left foot (maximum time 40 sec)  - Dynamic balance - jump sideways with feet together over a 25 × 10 cm platform attached to floor (total time in units of 0.1 sec) | test - retest reliability range from  r = .86 - .94 [122, 123] | N/A | 1 |
| BOT-2SF | **Bruininks-Oseretsky Test of Motor Proficiency-Version 2 Short Form (2005)** | 4 to 21 | 14 items | **Single Items:**  - Dropping and catching a ball (both hands)  - Dribbling a ball (alternating hands)  **Scale:** N/A | **Single Items:**  - One-legged stationary hop (only strong leg)  - Jumping in place (same sides synchronised)  **Scale:** N/A | **Single Items:**  - Walking forward on a line)  - One-legged stance on balance beam (eyes open)  **Scale:** N/A | inter-rater reliability >.90, test–retest reliability > .80, internal consistency >.80 [124] | evidence is provided for the content and construct validity [125] | 2 |
| MABC | **Movement Assessment Battery for Children (1992)** | 4 to 12 | 32 items:  4 age bands (each 8 items) | **Single Items:**  - Catching bean bag  - Rolling ball between goal posts  **Scale:** 0-5 per skill |  | **Single Items:**  - Standing on one leg  - Walking heels raised  - Jumping over cord  **Scale:** 0-5 per skill | test-retest reliability for 5-6 year old children range from  r = .92 - .98 [126] | excellent validity [125] | 1 |
| MABC-2 | **Movement Assessment Battery for Children-Version 2 (2007)** | 3 to 16 | 24 items:  3 age bands  (each 8 items) | **Single Items:**  - Catching bean bag  - Throwing bean bag onto  mat  **Scale:** 1-5 per skill |  | - **Single Items:**  - Standing on one leg  - Walking heels raised on a line  - Jumping on mats  **Scale:** 1-5 per skill | test–retest  reliability for 3-6 year old children range from r = .86 - .91 [127] | N/A | 1 |
| MOTB3-7 | **Motor Test Battery (2011)** | 3 to 7 | 10 items |  | **Single Items:**  - Hopping sideways over line (Jumps/30sec)  - Standing long jump (Distance in cm)  - Run (4x4m) (Time in sec)  **no Scale** | **Single Items:**  - Walking on balance beam (Number/correct steps)  - Walking backwards on balance beam (Number/correct steps)  **no Scale** | test-retest reliability  gross motor score: r = .65  for single items: r = .87 [128] | construct validity is assumed by author [128] | 1 |
| MOT4-6 | **Motorik Test für 4-6 Jährige (1987)** | 4 to 6 | 18 items | **Single Items:**  - Catch ring  - Throw tennis ball at target  **Scale:** 0-2 per skill | **Single Items:**  - Move 3 tennis balls from one box to another as fast as possible  - Move through a ring as fast as possible  - Jumping jacks  - Body roll to both sides with body fully stretched  **Scale:** 0-2 per skill | **Single Items:**  - Walking forward / backwards on a line  - One-legged hop in ring, hold for 5sec  - Stand up from sitting with legs crossed without arms  - Jumping in ring while turning 180°  - Jumping over cord  **Scale:** 0-2 per skill | test-retest reliability r = .85;  validity r = .68 [129] | criterion validity with „Körperkoordinationstest für Kinder“ (KTK) r = .68 [129] | 2 |
| LAP-3 | **Learning Achievement Profile 3^rd^ Edition (2004)** | 3 to 6 | 383 items; | Gross motor skills  **Scale:** 1-54 | | | test-retest reliability range from  r = .96 to .99 [130] | criterion validity with Battelle Developmental Inventory (BDI) Gross motor skills r = .81 [130] | 1 |
| ZNA3-5 | **(Adapted) Zurich Neuromotor Assessment Test 2012** | 3 to 5 | N/A |  | **Single Items:**  - Side-to-side jumping  - Hopping on one leg  - Forward jumping  - Walking on a straight line  - Walking on a bar  - Sitting up and down  - Running  - Climbing stairs  **Scale:** 1-5 per skill | **Single Items:**  - Standing on one foot  **Scale:** 1-5 per skill | test-retest reliability  Intra class correlation: r =0 .5, Intra class correlation: r = 0.8  of global motor score [131] | N/A | 1 |
| PDMS-2 | **Peabody Development Motor Scale 2^nd^ Edition (2000)** | birth to 6 | 249 items | **Single Items:**  e.g. catching, throwing, and kicking (24 items)  **Scale:** Subscale standard score 0-20 | **Single Items:**  e.g. crawling, walking, running, hopping, and jumping forward (89 items)  **Scale:** Subscale standard score 0-20 | **Single Items:**  balance (30 items)  **Scale:** Subscale standard score 0-20 | test–retest reliability on gross motor scale r=.98 [36] | criterion validity with PDMS Gross motor quotient r = .84 [132]  criterion validity with Mullen Scales of Early Learning – AGS Edition (MSEL:A) Gross motor quotient r = .86 [132] | 2 |
| TGMD | **Test of Gross Motor Development (1985)** | 3 to 10 | 12 items | **Single Items:**  - Striking stationary ball  - Stationary dribble  - Catch  - Kick  - Overhand throw  **Scale:** Subscale raw score 0-19 | **Single Items:**  - Run  - Gallop  - Hop  - Leap  - Horizontal jump  - Skip  - Slide  **Scale:** Subscale raw score 0-28 |  | split-half reliability coefficients ranging from r = .84 to .90 [37] | N/A | 6 |
| TGMD-2 | **Test of Gross Motor Development 2^nd^ Edition (2000)** | 3 to 10 | 12 items | **Single Items:**  - Striking stationary ball  - Stationary dribble  - Catch  - Kick  - Overhand throw  - Underhand roll  **Scale:** Subscale raw score 0-48, Subscale standard score 1-20 | **Single Items:**  - Run  - Gallop  - Hop  - Leap  - Horizontal jump  - Slide  **Scale:** Subscale raw score 0-48, Subscale standard score 1-20 |  | test-retest reliability  locomotion r = .85  object Control: r = .88  Gross Motor Composite: r = .91 in early childhood [8] | criterion validity with Basic Motor Generalizations subtest of the Comprehensive Scale of Student Abilities (CSSA) locomotor subscale r = .63, and object control subscale r = .41, composite score r = .63 [88] | 8 |

*m* meter, *N/A* not available, *sec* seconds

^a^ Three studies [42, 71, 72] used single items to asses fundamental movement skills (see Table 1).

^b^ All items of test batteries – table gives only an overview of object control, locomotor and balance items.

^c^ Numbers of included studies using the test batteries.
