# Supplementary material for: Interventions to Promote Fundamental Movement Skills in Childcare and Kindergarten: A Systematic Review and Meta-Analysis
Source: Sports Med. 2017 Apr 6;47(10):2045–68. doi: 10.1007/s40279-017-0723-1 (PMC5603621; doi:10.1007/s40279-017-0723-1)
Supplement: Supplementary file 7 — Electronic Supplementary Material Table S3 (DOCX 21 kb) [file 40279_2017_723_MOESM7_ESM.docx]

| **Electronic Supplementary Material Table S3.** Component ratings of studies | |
| --- | --- |
| For each of the eight components, use the following descriptions as a roadmap. | |
| **A) SELECTION BIAS** | |
| **Strong** | The selected individuals are likely to be representative of the target population (Q1 is 1); **and** the participation is 80% or more (Q2 is 1). |
| **Moderate** | The selected individuals are likely to be representative of the target population (Q1 is 1) **or** at least somewhat likely (Q1 is 2); **and** the participation is 60 – 79% (Q2 is 2) **or** can’t tell (Q2 is 5). |
| **Weak** | The selected individuals are not likely to be representative of the target population (Q1 is 3) **or** not described (Q1 is 4); **or** the participation is less than 60% (Q2 is 3). |
| **B) DESIGN** | |
| **Strong** | The study is described as RCT (Q1 is 1); **and** the exact description of randomisation is present (Q2-4 are Yes). |
| **Moderate** | The study is described as CT (Q1 is 2); **or** the study is described as RCT without exact description of randomisation (Q3 is No) **or** (Q4 is No). |
| **Weak** | No weak rating as all trials were CTs or RCTs. |
| **C) CONFOUNDERS^a^** | |
| **Strong** | There is an adjustment for 80% of relevant confounders or more (Q2 is 1). |
| **Moderate** | There are important differences between groups (Q1 is 1); **and** there is an adjustment for 60 – 79% of relevant confounders (Q2 is 2). |
| **Weak** | There are important differences between groups (Q1 is 1); **and** there is an adjustment for less than 60% of relevant confounders (Q2 is 3); **or** control of confounders was not described (Q1 is 3) **and** (Q2 is 4). |
| **D) BLINDING** | |
| **Strong** | No strong rating is possible, as parents/children/staff of childcares or kindergartens somehow know about the intervention (Q2 is always 1). |
| **Moderate** | The outcome assessors are not aware of the intervention status of participants (Q1 is 2). |
| **Weak** | The outcome assessors are aware of the intervention status of participants (Q1 is 1); **or** blinding of outcome assessors is not described (Q1 is 3). |
| **E) DATA COLLECTION METHODS** | |
| **Strong** | The data collection tools are valid (Q1 is 1); **and** the data collection tools are reliable (Q2 is 1). |
| **Moderate** | The data collection tools are either valid (Q1 is 1) **or** reliable [r≥0.7] (Q2 is 1). |
| **Weak** | The data collection tools are not valid (Q1 is 2); **and** the data collections tools are not reliable (Q2 is 2); or both validity and reliability are not described (Q1 is 3 and Q2 is 3). |
| **F) WITHDRAWALS AND DROP-OUTS** | |
| **Strong** | The follow-up rate is 80% or more (Q2 is 1). |
| **Moderate** | The follow-up rate is 60 – 79% (Q2 is 2); **and** the reason for withdrawal/drop-outs is reported (Q1 is 1). |
| **Weak** | The follow-up rate is less than 60% (Q2 is 3); **or** the follow-up rate is not described (Q2 is 4). |
| **G) INTERVENTION INTEGRITY** | |
| **Strong** | Measurement of consistency was made (Q2 is 1); **and** the number of participants receiving the intervention is 80% or higher (Q1 is 1); **and** no contamination occurred (Q3 is 2) **or** can’t tell (Q3 is 3). |
| **Moderate** | Measurement of consistency was made (Q2 is 1); **and** the number of participants receiving the intervention is 60 – 79% (Q1 is 2) **or** can’t tell (Q1 is 4); **and** no contamination occurred (Q3 is 2) **or** can’t tell (Q3 is 3). |
| **Weak** | No measurement of consistency was made (Q2 is 2) **or** can’t tell (Q2 is 3); **or** the number of participants receiving the intervention is less than 60% (Q1 is 3); **or** contamination may have occurred (Q3 is 1). |
| **H) ANALYSES^b^** | |
| **Strong** | All three statistical adjustments are made; **and** the analysis is performed by intention to treat (Q4 is 1). |
| **Moderate** | At least one out of three statistical adjustments is made; **and** the analysis is performed by intention to treat (Q4 is 1) **or** can’t tell (Q4 is 3). |
| **Weak** | No statistical adjustments are made; **or** the analysis is not performed by intention to treat (Q4 is 2). |
| *FMS* fundamental movement skills, *SES* socio-economic status  ^a^ We considered sex, age, SES, baseline FMS values, and cluster as relevant confounders.  ^b^ The focus in this component lies on three statistical adjustments: cluster adjustment, baseline adjustment, adjustment for age/sex/SES. | |
