# Supplementary material for: Interventions to Promote Fundamental Movement Skills in Childcare and Kindergarten: A Systematic Review and Meta-Analysis
Source: Sports Med. 2017 Apr 6;47(10):2045–68. doi: 10.1007/s40279-017-0723-1 (PMC5603621; doi:10.1007/s40279-017-0723-1)
Supplement: Supplementary file 8 — Electronic Supplementary Material Table S4 (DOCX 35 kb) [file 40279_2017_723_MOESM8_ESM.docx]

| **Electronic Supplementary Material Table S4.** Rating of studies | | | | | | | | | | | |  |
| --- | --- | --- | --- | --- | --- | --- | --- | --- | --- | --- | --- | --- |
| **Study** [ref.]*, Country | **Final decision** | Selection bias | Study design^b^ | Confounders | Blinding | Data collection methods | Withdrawal/  Dropouts | Intervention integrity | Analyses | **Methodological quality** | **Size of Study^c^** |  |
| **Randomized controlled trials^a^** | | | | | | | | | | | |  |
| Bonvin et al. (2013) [69], Switzerland | **1** | 1 | 2 | 1 | 2 | 1 | 1 | 2 | 1 | High | 1 |  |
| Donath et al. (2015) [38], Switzerland | **1** | 1 | 2 | 1 | 2 | 1 | 2 | 1 | 2 | High | 0 |  |
| Hardy et al. (2010) [40], Australia | **1** | 2 | 1 | 1 | 2 | 1 | 1 | 2 | 1 | High | 1 |  |
| Hurmeric (2011) [51], United States | **1** | 1 | 2 | 2 | 2 | 1 | 1 | 1 | 2 | High | 0 |  |
| Jones et al. (2011) [75], Australia | **1** | 1 | 1 | 2 | 2 | 1 | 1 | 2 | 1 | High | 0 |  |
| Puder et al. (2011) [71], Switzerland | **1** | 1 | 1 | 1 | 2 | 1 | 1 | 1 | 1 | High | 1 |  |
| Reilly et al. (2006) [45], Scotland | **1** | 2 | 1 | 1 | 2 | 1 | 1 | 2 | 1 | High | 1 |  |
| Roth et al. (2015) [72], Germany | **1** | 2 | 1 | 1 | 2 | 1 | 1 | 2 | 1 | High | 1 |  |
| Alhassan et al. (2012) [67], United States | **2** | 2 | 2 | 2 | 2 | 1 | 1 | 1 | 2 | Moderate | 0 |  |
| Bellows et al. (2013) [36], United States | **2** | 2 | 2 | 1 | 3 | 1 | 2 | 2 | 2 | Moderate | 1 |  |
| Zask et al. (2012) [49], Australia | **2** | 1 | 2 | 1 | 3 | 1 | 2 | 2 | 1 | Moderate | 1 |  |
| Derri et al. (2001) [70], Greece | **3** | 3 | 2 | 2 | 3 | 1 | 3 | 3 | 2 | Low | 0 |  |
| Piek et al. (2013) [44], Australia | **3** | 1 | 2 | 3 | 3 | 1 | 2 | 2 | 2 | Low | 1 |  |
| Vidoni et al. (2014) [68], United States | **3** | 3 | 2 | 2 | 3 | 1 | 3 | 3 | 3 | Low | 0 |  |
| **Controlled trials^a^** | | | | | | | | | | | |  |
| Goodway & Branta (2003) [60], United States | **2** | 2 | 2 | 2 | 2 | 1 | 2 | 2 | 3 | Moderate | 0 | |
| Goodway et al. (2003) [61], United States | **2** | 2 | 2 | 2 | 3 | 1 | 2 | 1 | 2 | Moderate | 0 | |
| Krombholz (2012) [43], Germany | **2** | 2 | 2 | 1 | 3 | 1 | 2 | 2 | 2 | Moderate | 1 | |
| Robinson& Goodway (2009) [55], United States | **2** | 2 | 2 | 2 | 2 | 1 | 1 | 1 | 2 | Moderate | 1 |  |
| Yin et al. (2012) [52], United States | **2** | 1 | 2 | 2 | 3 | 1 | 1 | 2 | 2 | Moderate | 1 |  |
| Deli et al. (2006) [37], Greece | **3** | 2 | 2 | 2 | 2 | 1 | 1 | 3 | 3 | Low | 0 |  |
| Hamilton et al. (1999) [39], United States | **3** | 2 | 2 | 2 | 2 | 1 | 3 | 3 | 2 | Low | 0 |  |
| Hashemi et al. (2015) [74], Iran | **3** | 2 | 2 | 3 | 3 | 1 | 3 | 3 | 3 | Low | 0 |  |
| Ignico (1991) [41], United States | **3** | 3 | 2 | 3 | 3 | 1 | 3 | 3 | 2 | Low | 0 |  |
| Iivonen et al. (2011) [54], Finland | **3** | 2 | 2 | 2 | 3 | 1 | 3 | 3 | 1 | Low | 0 |  |
| Kelly et al. (1989) [42], United States | **3** | 2 | 2 | 3 | 3 | 1 | 3 | 2 | 2 | Low | 0 |  |
| Tsapakidou et al. (2014) [46], Greece | **3** | 3 | 2 | 3 | 3 | 1 | 1 | 3 | 3 | Low | 0 |  |
| Valentini (1999) [62], United States | **3** | 2 | 2 | 2 | 3 | 1 | 1 | 3 | 2 | Low | 0 |  |
| Venetsanou & Kambas (2004) [73], Greece | **3** | 3 | 2 | 2 | 3 | 1 | 3 | 3 | 3 | Low | 0 |  |
| Wang (2004) [47], Taiwan | **3** | 2 | 2 | 3 | 3 | 1 | 3 | 3 | 3 | Low | 0 |  |
| Weiss et al. (2004) [48], Germany | **3** | 3 | 2 | 3 | 3 | 1 | 3 | 3 | 3 | Low | 0 |  |
| * Reference number from manuscript  ^a^ As described by the authors of the study  ^b^ As rated according to the quality assessment criteria  ^c^ For n participants < 100, size of study = 0; For n participants ≥ 100, size of study = 1 | | | | | | | | | | | |  |
