# Supplementary material for: Interventions to Promote Fundamental Movement Skills in Childcare and Kindergarten: A Systematic Review and Meta-Analysis
Source: Sports Med. 2017 Apr 6;47(10):2045–68. doi: 10.1007/s40279-017-0723-1 (PMC5603621; doi:10.1007/s40279-017-0723-1)
Supplement: Supplementary file 9 — Electronic Supplementary Material Table S5 (DOCX 36 kb) [file 40279_2017_723_MOESM9_ESM.docx]

| **Electronic Supplementary Material Table S5.** Studies examining the effect of intervention | | | | | | | |
| --- | --- | --- | --- | --- | --- | --- | --- |
| **Methodological Quality** | **Study**  **[reference number]** | **Abbreviation FMS Test** | **FMS Outcomes measure** | **Adjustment for age (y/n)**** | **% (pre-post); SMD_within_ CON (95% CI)** | **% (pre-post); SMD_within_ INT (95% CI)** | **SMD_between_ INT vs. CON (95% CI)** |
|  |  |  |  |  |  |  |  |
| high | Bonvin et al. 2013 [69] | ZNA3-5 | Global motor score | y | CON: +12.0% (↑); 0.57 (0.41, 0.72) | INT: +12.7% (↑); 0.56 (0.40, 0.72) | 0.00 (-0.16, 0.16) |
| high | Donath et al. 2015 [38] | TGMD-2 | OCS raw score* | n | CON: +11.5% (↑); 0.22 (-0.18, 0.61) | INT: +21.6% (↑); 0.50 (0.26, 0.73) | 0.48 (-0.15, 1.11) |
| high | Hardy et al. 2010 [40] | TGMD-2 | LMS raw score |  | CON: +3.6% (↑); 0.12 (-0.09, 0.34) | INT: +8.3% (↑); 0.31 (0.14, 0.48) | 0.46 (0.24, 0.68) |
|  |  |  | OCS raw score | n | CON: +8.2% (↑); 0.30 (0.08, 0.51) | INT: +12.3% (↑); 0.48 (0.30, 0.65) | 0.37 (0.15, 0.59) |
|  |  |  | Total FMS score |  | CON: +5.4% (↑); 0.24 (0.03, 0.46) | INT: +9.8% (↑); 0.46 (0.29, 0.63) | 0.53 (0.31, 0.75) |
| high | Hurmeric 2011 [51] | TGMD-2 | OCS standard score | y | CON: +3.4% (↑); 0.14 (-0.41, 0.70) | INT: +36.6% (↑); 2.42 (1.63, 3.22) | 2.79 (1.97, 3.61) |
| high | Jones et al. 2011 [75] | TGMD-2 | Total standard score (5 items) | y | CON: +11.0% (↑); 0.37 (-0.04, 0.79) | INT: +23.0% (↑); 0.84 (0.44, 1.24) | 0.42(-0.01, 0.85) |
| high | Puder et al. 2011 [71] | Single items | mean value of four single items* | y | CON: +20.0% (↑); 0.60 (0.37, 0.84) | INT: +21.7% (↑); 0.61 (0.16, 1.05) | 0.07 (-0.07, 0.22) |
| high | Reilly et al. 2006 [45] | MABC | Fundamental movement score | y | CON: +28.0% (↑); 1.15 (0.97, 1.33) | INT: +31.3% (↑); 1.46 (1.27, 1.66) | 0.33 (0.15, 0.51) |
| high | Roth et al. 2015 [72] | Single items | Composite z-score | y | *N/A* | *N/A* | 0.62 (0.28, 0.96) |
| moderate | Alhassan et al. 2012 [67] | TGMD-2 | LMS percentile score | y | CON: +32.6% (↑); 0.64 (0.13, 1.14) | INT: +42.9% (↑); 1.17 (0.73, 1.62) | 0.56 (0.07, 1.05) |
| moderate | Bellows et al. 2013 [36] | PDMS-2 | Balance subscale standard score | y | CON: -6.3% (↓); -0.27 (-0.51, -0.03) | INT: +8.3% (↑); 0.44 (0.19, 0.68) | 0.60 (0.33, 0.87) |
|  |  |  | LMS standard score | y | CON: +3.1% (↑); 0.17 (-0.07, 0.41) | INT: +11.5% (↑); 0.65 (0.40, 0.89) | 0.41 (0.14, 0.68) |
|  |  |  | OCS standard score | y | CON: +1.3% (↑); 0.06 (-0.18, 0.31) | INT: +9.5% (↑); 0.43 (0.19, 0.68) | 0.53 (0.26, 0.80) |
|  |  |  | Gross motor quotient | y | CON: -0.5% (↓); -0.06 (-0.30, 0.18) | INT: +5.7% (↑); 0.62 (0.37, 0.87) | 0.68 (0.41, 0.95) |
| moderate | Goodway & Branta 2003 [60] | TGMD | LMS raw score (percentile) | y | CON: +14.1% (↑); 0.81 (0.27, 1.36) | INT: +48.5% (↑); 3.75 (2.91, 4.59) | 2.76 (2.03, 3.49) |
|  |  |  | OCS raw score (percentile) | y | CON: +57.5% (↑); 1.79 (1.17, 2.42) | INT: +75.8% (↑); 4.34 (3.40, 5.27) | 2.14 (1.49, 2.79) |
| moderate | Goodway et al. 2003 [61] | TGMD | OCS raw score (percentile) | y | CON: 15.4% (↑); 0.22 (-0.28, 0.73) | INT: 69.2% (↑); 2.33 (1.67, 3.00) | 1.73 (1.14 2.31) |
|  |  |  | LMS raw score (percentile) | y | CON: 1.1% (↑); 0.03 (-0.47, 0.54) | INT: 46.4% (↑); 2.51 (1.86, 3.17) | 2.06 (1.44, 2.68) |
| moderate | Krombholz 2012 [43] | MOTB 3-7 | Total Performance score | y | CON: N/A; 0.25 (0.06, 0.44) | INT: N/A; 0.60 (0.40, 0.79) | 0.44 (0.24, 0.64) |
| moderate | Robinson & Goodway 2009 [55] | TGMD-2 | LMS raw score (percentile) | y | *N/A* | *N/A* | 3.42 (2.83, 4.01) |
| moderate | Yin et al. 2012 [52] | LAP-3 | Gross motor development raw score | n | CON: +20.9% (↑); 0.82 (0.60, 1.03) | INT: +18.4% (↑); 0.90 (0.68, 1.12) | 0.74 (0.43, 1.05) |
| moderate | Zask et al. 2012 [49] | TGMD-2 | LMS raw score |  | CON: +11.0 % (↑); 0.94 (0.70, 1.19) | INT: +11.1% (↑); 1.42 (1.23, 1.61) | 0.44 (0.24, 0.64) |
|  |  |  | Movement skills quotient score | y | CON: +4.1% (↑); 0.47 (0.23, 0.71) | INT: +14.2% (↑); 1.28 (1.09, 1.47) | 0.54 (0.32, 0.76) |
| low | Deli et al. 2006 [37] | TGMD | *N/A* | *N/A* | *N/A* | *N/A* | *N/A* |
| low | Derri et al. 2001 [70] | TGMD | LMS raw score* | n | CON: +13.8% (↑); 0.40 (0.21, 0.58) | INT: +22.4% (↑); 0.71 (0.58, 0.85) | 0.62 (0.13, 1.11) |
| low | Hamilton et al. 1999 [39] | TGMD | OCS standard score | y | CON: -16% (↓); -0.25 (-1.05, 0.55) | INT: +46.3% (↑); 1.60 (0.76, 2.44) | 2.09 (1.13, 3.05) |
| low | Hashemi et al. 2015 [74] | TGMD-2 | OCS raw score* | n | CON: +3.2% (↑); 0.10 (-0.11, 0.30) | INT: +29.5% (↑); 1.19 (0.96, 1.41) | 1.14 (0.59, 1.69) |
| low | Ignico 1991 [41] | TGMD | Gross motor development quotient | y | CON: -2.3% (↓); -0.13 (-0.84, 0.59) | INT: +15.4% (↑); 1.23 (0.44, 2.02) | 1.73 (0.87, 2.59) |
| low | Iivonen et al. 2011 [54] | APM Inventory | Object control skill score | *N/A* | CON: +17.6% (↑); 0.56 (0.09, 1.04) | INT: +24.5% (↑); 0.79 (0.33, 1.25) | 0.41 (-0.02, 0.84) |
| low | Kelly et al. 1989 [42] | Single items | Six items | *N/A* | *N/A* | *N/A* | *N/A* |
| low | Piek e al. 2013 [44] | BOT-2SF | Total standard score | y | CON: 0% (↔); 0.02 (-0.17, 0.22) | INT: +3.9% (↑); 0.23 (0.06, 0.41) | -0.11 (-0.31, 0.09) |
| low | Tsapakidou et al. 2014 [46] | TGMD-2 | LMS standard score | y | CON: +11.0% (↑); 0.44 (0.03, 0.84) | INT: +32.1% (↑); 1.53 (1.08, 1.98) | 1.13 (0.70, 1.56) |
| low | Valentini 1999 [62] | TGMD | OCS standard score | y | CON: +16.9% (↑); 0.94 (0.39, 1.48) | INT: +35.3% (↑); 2.30 (1.71, 2.98) | 0.34 (-0.15, 0.83) |
|  |  |  | LMS standard score | y | CON: +45.9% (↑); 3.70 (2.84, 4.57) | INT: +55.3% (↑); 4.08 (3.27, 4.88) | 0.82 (0.31, 1.33) |
| low | Venetsanou & Kambas 2004 [73] | MOT4-6 | Total raw score | n | CON: +5.9% (↑); 0.19 (-0.26, 0.64) | INT: +20.4% (↑); 0.70 (0.16, 1.24) | 0.64 (0.13, 1.15) |
| low | Vidoni 2014 [68] | BOT-2SF | Total standard score | y | CON: +7.1% (↑); 0.41 (-0.32, 1.13) | INT: +18.0% (↑); 1.13 (0.42, 1.84) | 0.50 (-0.21, 1.21) |
| low | Wang 2004 [47] | PDMS-2 | *N/A* | *N/A* | *N/A* | *N/A* | *N/A* |
| low | Weiss  et al. 2004 [48] | MOT4-6 | *N/A* | *N/A* | *N/A* | *N/A* | *N/A* |

*CI* confidence interval, *CON* control group, *FMS* fundamental movement skills, *INT* intervention group, *LMS* locomotor subscale, *n* no, *N/A* not available, *OCS* object control subscale, *SMD* standardized mean difference, *y* yes

* Weighted SMD of all single items. A total or subscale score was not available.

** FMS outcome measures are scaled scores or percentiles for age categories based on half-yearly or yearly steps to adjust for age and maturational effects.
